# Implementation of Contraction to Electrophysiological Ventricular Myocyte Models, and Their Quantitative Characterization via Post-Extrasystolic Potentiation

**DOI:** 10.1371/journal.pone.0135699

**Published:** 2015-08-28

**Authors:** Yanyan Claire Ji, Richard A. Gray, Flavio H. Fenton

**Affiliations:** 1 Department of Physics, Georgia Institute of Technology, Atlanta, Georgia, United States of America; 2 Office of Science and Engineering Laboratories, Center for Devices and Radiological Health, Food and Drug Administration, Silver Spring, Maryland, United States of America; Temple University, UNITED STATES

## Abstract

Heart failure (HF) affects over 5 million Americans and is characterized by impairment of cellular cardiac contractile function resulting in reduced ejection fraction in patients. Electrical stimulation such as cardiac resynchronization therapy (CRT) and cardiac contractility modulation (CCM) have shown some success in treating patients with HF. Computer simulations have the potential to help improve such therapy (e.g. suggest optimal lead placement) as well as provide insight into the underlying mechanisms which could be beneficial. However, these myocyte models require a quantitatively accurate excitation-contraction coupling such that the electrical and contraction predictions are correct. While currently there are close to a hundred models describing the detailed electrophysiology of cardiac cells, the majority of cell models do not include the equations to reproduce contractile force or they have been added ad hoc. Here we present a systematic methodology to couple first generation contraction models into electrophysiological models via intracellular calcium and then compare the resulting model predictions to experimental data. This is done by using a post-extrasystolic pacing protocol, which captures essential dynamics of contractile forces. We found that modeling the dynamic intracellular calcium buffers is necessary in order to reproduce the experimental data. Furthermore, we demonstrate that in models the mechanism of the post-extrasystolic potentiation is highly dependent on the calcium released from the Sarcoplasmic Reticulum. Overall this study provides new insights into both specific and general determinants of cellular contractile force and provides a framework for incorporating contraction into electrophysiological models, both of which will be necessary to develop reliable simulations to optimize electrical therapies for HF.

## Introduction

Heart failure (HF) affects over 5 million Americans and is characterized by impairment of cellular cardiac contractile function and reduced ejection fraction in patients[1]. Electrical stimulation such as cardiac resynchronization therapy (CRT) and cardiac contractility modulation (CCM) have shown success in treating patients with HF[2,3].

CRT is a current treatment for patients with congestive heart failure (CHF) for a wide QRS complex, where biventricular pacemakers are used to improve electrical synchrony and presumably ejection fraction[2,4]. However, the response to CRT varies greatly among patients. Improvements are quite variable with up to 30% of patients being non-responders to this treatment at all[2,5]. Nevertheless, the efficacy and optimization of CRT continues to being improved[6,7]. For example, it has been shown that extensive electrical remodeling is significantly associated with better survival rates after CRT[6]. Other measurements than electrical dyssynchrony have been investigated as identifications for CRT implantation estimation. It has been suggested that mechanical dyssynchrony is as well essential in prediction of CRT response[7].

An alternate electrical therapy for HF is cardiac contractility modulation (CCM) which delivers electric fields to the heart during its refractory period. Although its mechanism of action was initially thought to result from increased calcium flux from Sarcoplasmic Reticulum (SR)[3,8], recent studies suggest this is not the case, but that increased contractile function is the result of beta adregenergic stimulation[9]. Clinical studies have showed that CCM therapy improved quality of life, exercise capacity, New York Heart Association (NYHA) class, and ejection fraction (EF) during long-term follow up[10]. However, other clinical studies have reported contradictory evidence where CCM did not change effects on hospitalization or mortality[11], and it did not increase myocardial oxygen consumption[12].

It is clear then, that optimization of CRT and CCM therapies require an understanding of the effects of electric fields on myocyte dynamics especially for inotropicity as regulated by intracellular calcium. Computer simulations have the potential to improve these therapies through clarification of the underlying mechanisms[13]. For example, one challenge in CRT is to determine the optimal pacing locations; Miri et al. provided an optimization strategy to find the best pacing sites and timing delays in CRT based on biventricular-paced activation sequences and ECGs obtained from simulations using patient-specific anatomy and pathophysiology models[14]. Computer simulations can also be beneficial to help to predict the response of patients to certain therapies[15,16]. For instance, Niederer et al. have designed an electromechanical heart model based on clinical observations[17]. The model predicted that patients with dyssynchronous electrical activation but effective length-dependent tension regulation at the cellular scale are less likely to respond to CRT treatment than patients with attenuated or no length dependent of tension.

Such simulations of electrical therapy require a robust model of cellular excitation-contraction coupling that occurs in the cardiac myocyte such that the electrical and contraction predictions are *both* accurate. During the depolarization of an action potential, L-type *Ca* ^2+^ channels are activated and the influx of *Ca* ^2+^ current into the narrow dyadic space induces a large *Ca* ^2+^ release from the Junctional Sarcoplasmic Reticulum (JSR) through the Ryanodine Receptor (RyR). Intracellular *Ca* ^2+^ concentration is thus greatly increased immediately following the depolarization of the transmembrane action potential. Some of these *Ca* ^2+^ ions bind to affinity sites on Troponin C therefore enable myosin heads, which contain cross-bridges, to attach to actin. Myosin heads ‘walk’ on actin generating force, transforming chemical energy to mechanical energy resulting in cell contraction. Then during the repolarization of the action potential, *Ca* ^2+^ leaves the cytoplasmic compartment into either the Network Sarcoplasmic Reticulum (NSR) through the Ca-ATPase pump or out of the cell membrane though Na-Ca exchangers. The decreased intracellular *Ca* ^2+^ concentration causes *Ca* ^2+^ to detach from troponin C, inducing uncoupling of Myosin heads and actin, which ends the contraction process[18]. From this *Ca* ^2+^ handling process, we can see that *Ca* ^2+^ is deeply involved in the two highly non-linear systems, namely the electrical activation and tension generation and *Ca* ^2+^ binding with troponin C is the key component linking electrical signals to the activation of tension[18].

While there are close to a hundred cell models describing the detailed electrophysiology (EP) of cardiac cells, the majority of cell models do not include the equations to reproduce contractile force[19,20]. This is a considerable drawback. We suggest the most appropriate and challenging test for Electrical-contractile coupled (ECC) cell models derived from EP models is to reproduce the “postextrasystolic potentiation” (PESP) behavior of the heart[21]. PESP is an example of the changes in stimulation pattern on contractile strength. The effect can be demonstrated with the PESP pacing protocol[22]. First, a train of stimuli is delivered with a fixed basic cycle length called the priming period (PP). Second, an extrasystolic (ES) beat is delivered after the last priming stimulus by an interval called extrasystolic interval (ESI). Following the ES beat, a postextrasystolic (PES) beat is then delivered after another interval called the postextrasystolic interval (PESI). In normal hearts the strength of postrasystles is a function of both ESI and PESI. If ESI is fixed, postexatrasystolic strength increases as PESI is lengthened. If PESI is fixed, postextrasystolic strength increases as ESI is shortened. This effect includes mechanical restitution and pause dependence and it captures the important heart dynamics involving force generation and calcium cycling that occurs over multiple beats. Just like the electrical restitution protocol is a stringent test for electrical rate dependence, we believe that reproducing the PESP protocol is essential for an electromechanical cell model.

In this paper, we first present a systematical methodology to incorporate mechanical models into various existing EP models. We chose fourteen of the most recently developed EP models with multiple cellular compartments, advanced ionic currents and more realistic subcellular dynamics because they include the essential elements required to couple contraction models (e.g., SR dynamics and calcium buffers). We studied these models under the isometric condition by 1) evaluating how their calcium dynamics are affected by the inclusion of the contraction and 2) assessing how well these electromechanical models simulate contraction by comparing their PESP contractile response with experiments from Yue et al. and 3) analyzing the mechanism of PESP by finding the correlation between the calcium release from SR and the contractile strength. Finally we concluded with insights regarding the suitability of various models to reproduce electromechanical activities and the underlying mechanism of PESP, which may provide guidelines for future experiments.

## Methods

In this section we introduced the contraction model that we implemented to EP models and the experiment we compared our simulations to, then we described our classification of EP models and corresponding strategy for contraction implementation, the numerical methods used to solve the models as well as the pacing protocols and data analysis. All terminology can be found in Table 1

**Table 1:** Terminology: definition of variables.

Variable Type	Variable Name	Definition
Pacing intervals	SSI (PI,PP)	Steady state interval (or priming interval/priming period): interval between steady state stimuli. It's 500ms in our simulations
	ESI	Extrasystolic interval: interval between extrasystolic and the last steady state stimuli
	PESI	Postextrasystolic interval: interval between postextrasystolic and extrasystolic stimuli
Measured quantity*	dP/dt_max_(SS)	Maximum pressure rising rate of steady state beats
	dP/dt_max_(ES)	Maximum pressure rising rate of extrasystolic beats
	dP/dt_max_(PES)	Maximum pressure rising rate of postextrasystolic beats
	[Ca^2+^]_i_	Intracellular calcium concentration
Force-interval relations	MRC_pes_	Postextrasystolic mechanical restitution curve: normalized dP/dt_max_ of postextrasystoles plotted vs PESI
	MRC_es_	Extrasystolic mechanical restitution curve: normalized dP/dt_max_ of extrasystoles plotted vs ESI
	PESPC	Postextrasystolic potentiation curve: normalized dP/dt_max_ of fully restituted postextrasystoles (CR_max,pes_) plotted vs ESI
Parameters for MRC_pes_ **	CR_max,pes_	Maximum postextrasystolic contractile response: plateau value fpr MRC_pes_
	T_mrc,pes_	Time constant for MRC_pes_
	t_o,pes_	PESI-axis intercept value
Parameters for PESPC	A	Amplitude
	B	Plateau value
	T_pespc_	Time constant

* Yue et al 1985 paper measured pressure changing rate. In our simulations we calculate tension changing rate and we replace P with F

** Paramters for MRC_es_ are identical to MRC_pes_ except replacing the subtitle 'pes' with 'es'

### The Contraction Model and Experiment

We chose the **Negroni_Lascano_1996 (NL96)**[23]contraction model to represent the coupling of cross-bridge dynamics and intracellular *Ca* ^2+^ kinetics.

The muscle unit structure and cross-bridge dynamics are shown in Fig 1 in Negroni et al[23]. The muscle unit is composed of an inflexible thick filament (Myosin), a thin filament (actin) and an elastic paralleled element (titin). The cross-bridges are attached to the thick element on one end and can slide on the thin element on the other end. The total force is contributed by two parts: force generated from the elastic element (*F*_*p*_) and force generated by the cross-bridges (*F*_*b*_):

$$\begin{array}{l} F=F_{b}+F_{p}\\ F_{p}=K{\left(L-L_{0}\right)}^{5}\\ F_{b}=A([{TCa}^{*}]+[T^{*}])\text{h} \end{array}$$ (1)

where *K*, *A* and *L*_0_ are constants; *L* is the sarcomere length; TCa* and T* are two states associated with troponin C sites on the thin filament (defined below); *h* is the elongation of the muscle unit. For “isometric” contraction where the sarcomere length is fixed, *F*_*p*_ is constant, and the total force is determined only by the force generated from the cross-bridges. Cross-bridge grabbing and sliding on the thin element will generate force *F*_*b*_ which is proportional to the elongation (*h*) and the total number of attached cross-bridges. The number of attached cross-bridges is determined by the interaction with *Ca* ^2+^ and the relevant buffers, which is represented by a “four-state system” comprised of:1) sites on the thin element with free troponin C (*T*); 2) sites with *Ca* ^2+^ bound to troponin C (*TCa*); 3) sites with *Ca* ^2+^ bound to troponin C and attached cross-bridges (TCa*); and 4) sites with troponin C not bound to *Ca* ^2+^ but attached cross-bridges (T*). The system transitions among these four states via the binding and releasing of *Ca* ^2+^ and attaching and detaching of cross-bridges (See Fig 2 from Negroni et al [23]). The number of total cross-bridges is the sum of the two states that associated with cross-bridges ([TCa*]+[T*]). Equations represent the transitions among the four states in Fig 2 from Negroni et al [23] are:

$$\frac{d[T^{*}]}{dt}=(Y3[{TCa}^{*}]-\text{Z}3[{\text{T}}^{*}]{\left[Ca^{2+}\right]}_{i})-Y4[T^{*}]-Yd{\left(dX\;/\;dt\right)}^{2}[T^{*}]$$ (2)

$$\frac{d[TCa]}{dt}=(Y1{\left[Ca^{2+}\right]}_{i}[\text{T}]-\text{Z}1[\text{TCa}])-(\text{Y}\;2{\left[\text{TCa}\right]}_{eff}-\text{Z}\;2[{\text{TCa}}^{*}])$$ (3)

$$\frac{d[{TCa}^{*}]}{dt}=(Y2{\left[TCa\right]}_{eff}-\text{Z}\;2[{\text{TCa}}^{*}])-(Y\;3[{TCa}^{*}]-\text{Z}\;3[{\text{T}}^{*}]{\left[Ca^{2+}\right]}_{i})-Yd{\left(dX\;/\;dt\right)}^{2}[{TCa}^{*}]$$ (4)

$$\left[T]={\left[Troponin\right]}_{total}-[T^{*}]-[TCa]-[{TCa}^{*}\right]$$ (5)

**Fig 1:**
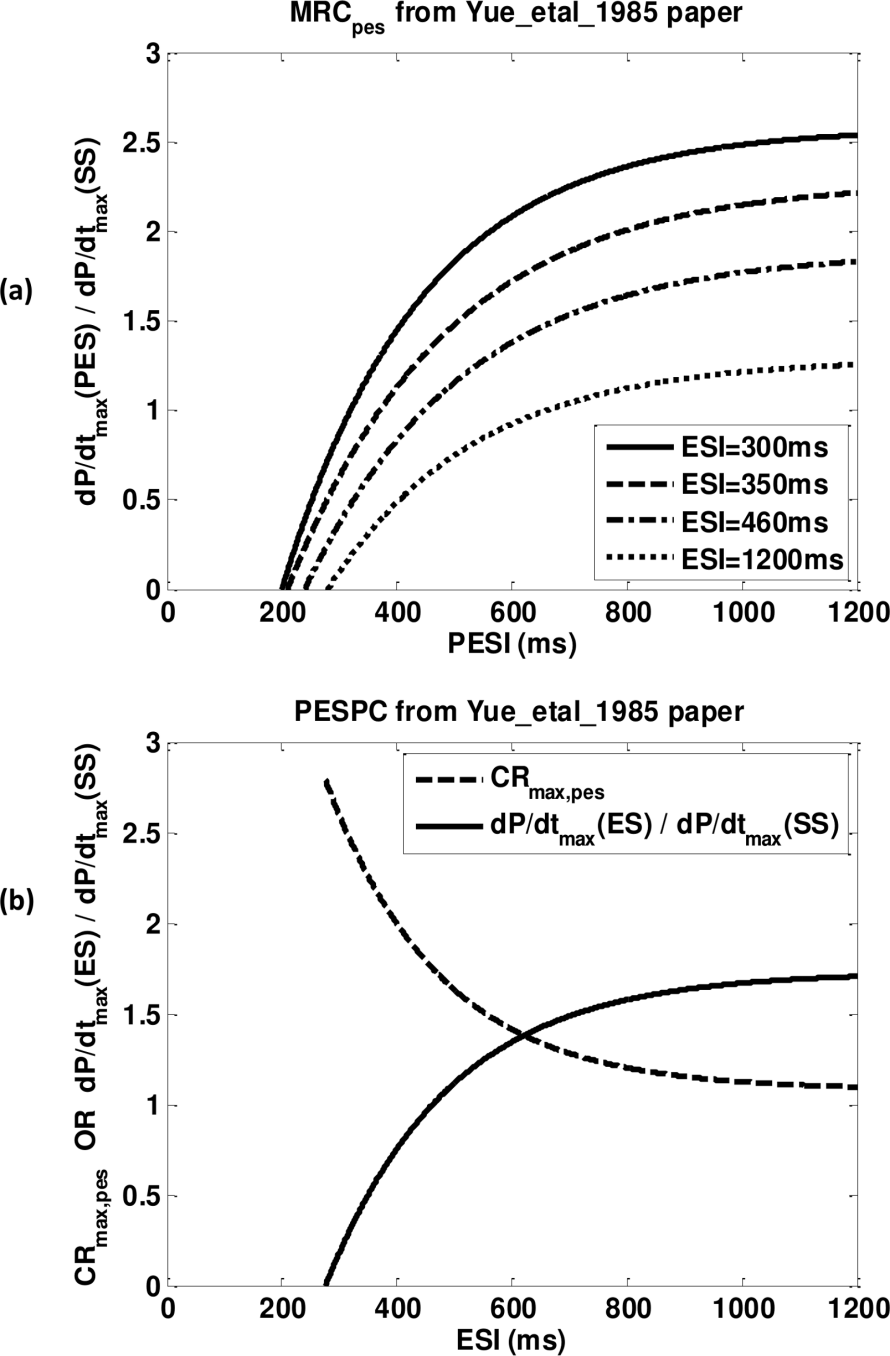
Contractile characteristic curves generated from equations in Yue experiments. (a) Postextrasystolic mechanical restitution curves (*MRC*_*pes*_) using ESI equal to 300ms (solid line), 350ms (dash line), 460ms (dash-dot line) and 1200ms (dot line). (b) Postextrasystolic potnetiation curve (PESPC, dash line) and extrasystolic mechanical restitution curve (*MRC*_*es*_, solid line).

**Fig 2:**
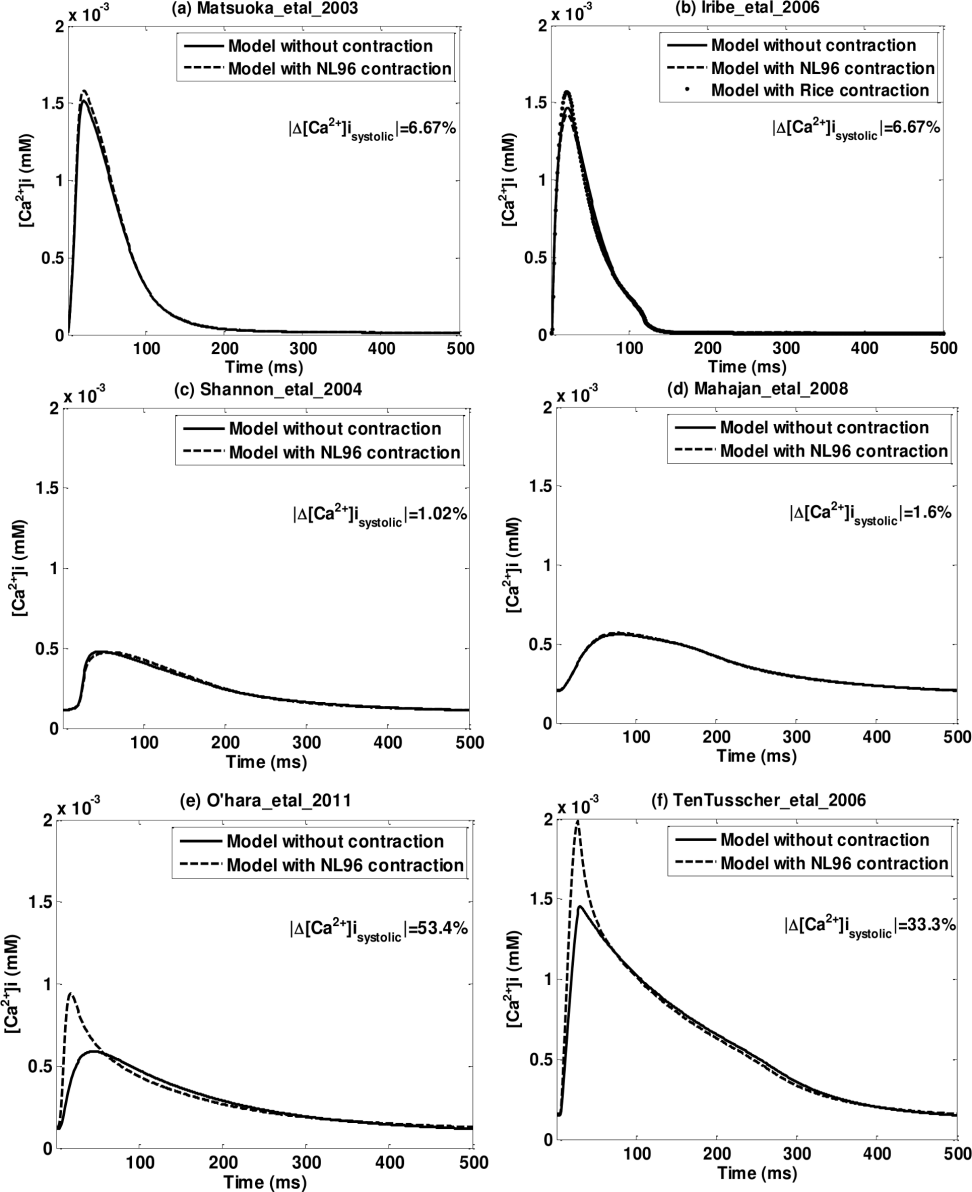
Priming [*Ca* ^2+^]_*i*_ transient for six representative models. (a) Matsuoka_etal_2003 (Type One: with contraction (NL96)); (b) Iribe_etal_2006 (Type One: with contraction (RWH99)); (c) Shannon_etal_2004 (Type Three: with full dynamic calcium buffers); (d) Mahajan_etal_2008 (Type Three: with dynamic calcium troponin buffer); (e) O’hara_etal_2011 (Type Four: with instantaneous calcium troponin buffer); (f) TenTusscher_etal_2006 (Type Five: with no calcium troponin buffer). Solid lines are models without contraction; dash lines are models with NL96 contraction. All figures have the same ranges in x and y axis. The insets show the relative difference in systolic [*Ca* ^2+^]_*i*_ between models with contractions and without contractions.

These are reaction equations among the states of free troponin (*T*), troponin attached with cross-bridges (T*), troponin bound with calcium (*TCa*), and troponin bound with calcium attached with cross-bridges (TCa*); all with units of mM/ms. [*TCa*]_*eff*_ is the effective [*TCa*] where cross-bridge attachment can occur and it’s dependent on the sarcomere length (see Eq 10 in [23]). *X* = *L* − *h*, which is the inextensible length in the muscle unit. *Y*1 ∼ *Y*4, *Z*1 ∼ *Z*3 and *Yd* are rate constants. The NL96 model includes feedback from force on *Ca* ^2+^; therefore it is, in general, a “strongly coupled” contraction model, but under isometric condition it is “weakly coupled” since *dX* / *dt* = 0 [24].

There are two reasons why we chose the NL96 model. First, it shares common elements with the EP models; this greatly simplifies the implementation procedure. For example, the four states in the *Ca* ^2+^ kinetics are closely related to intracellular *Ca* ^2+^ concentration and Troponin concentration, both of which are common elements in advanced EP models. Second, even though NL96 model is a simplified model of the average effect of the interaction between myosin and actin, it can effectively reproduce basic physiological findings, such as time course of isometric force, intracellular *Ca* ^2+^ transient and force-length- *Ca* ^2+^ relation. Third, our method to incorporate NL96 can be easily applied to other contraction models as long as they use ODEs to describe the *Ca* ^2+^ troponin states, like [25][26][27].

To evaluate the ability of these fourteen “coupled” electromechanical models to reproduce experimental findings, we compared the results of the simulations with the **isovolumetric canine ventricular experiments from Yue et al**.[22]. We chose Yue et al. because their paper includes not only a comprehensive experimental exploration of the relation between the contractile strength and pacing intervals but also a concise mathematical framework that allows quantitative comparisons of model predictions.

We applied the same PESP pacing protocol employed by Yue et al. [22], which was introduced in the Introduction section. By fixing ESI and varying PESI, Yue et al. [22] measured the rate of pressure change of the PES beat as a function of PESI; then by changing ESI to another value and repeatedly varying PESI, a family of curves can be constructed showing the effect of both ESI and PESI on pressure change (see Fig 2 from Yue et al [22]). In contrast to electrical restitution in which action potential duration is recorded for a single extrasystolic beat, the purpose of the PESP protocol is to study the contractile strength as a function of a single extrasystolic followed by a compensatory pause. The idea is to capture the full cycle *Ca* ^2+^ handling, specifically the release of calcium from the SR as a function of timing and the dynamics of the restoration of releasable SR *Ca* ^2+^ during (and following) action potential repolarization. The basic theory lies in the fact that *Ca* ^2+^ release from the SR is determined by two factors: the recovery of Ryanodine Receptor’s (RyR’s) which is a function of interbeat interval; and the amount of *Ca* ^2+^ in the SR. When ESI>PP, RyRs are more recovered from the last priming beat, resulting in a bigger *Ca* ^2+^ release from SR for the ES beat. Consequently, the *Ca* ^2+^ content in SR will be smaller compared to the priming period, and may lead to a smaller or larger *Ca* ^2+^ release (compared to PP) for the next PES beat depending on the PESI.

In Yue’s experiments, they measured the maximum rate of pressure change in fourteen isolated perfused left canine ventricles under isovolumetric conditions. In the NL96 contraction model, force (normalized to muscle cross section area) is the variable to quantify the contractile strength; therefore to correlate with Yue’s experiment results, we assumed a linear relationship between force generated from single cell and ventricular pressure[28], and thus we compare the normalized maximum rate of force change in our simulations with the normalized maximum rate of pressure change in Yue’s paper.

### Ventricular models and electrophysiological contraction implementation

We studied fourteen ventricular cell models, which include four species: guinea pig (n = 4)[29][30][31][32], rabbit (n = 2)[33][34], dog (n = 2)[35][36] and human (n = 6)[37][38][39][40][41][42]. We classify the models into five categories based on the type of their calcium buffers and designed corresponding strategies to implement NL96. The complexity of the implementation of contraction into these five types of models is listed in ascending order. The flowchart in the S1 File illustrates steps of the implementation. We provide the essential equations, initial conditions and choice of parameters below. More details can be found in the S1 File. Table 2 lists general information about all models including classification, species, buffer information, number of variables, stimulus current amplitudes and durations; rate constants for *Ca* ^2+^ troponin buffers can be found in Table 3.

**Table 2:** General information of 14 electrophysiological models including classification of models (model type, model name, species, type of intracellular *Ca* ^2+^ buffers) and model properties (number of variables in the original model, stimulus current amplitude and duration).

Model Type	Model Name	Species	CaTRPN buffer	Other Ca buffers	Number of variables	stimulus current(A/F)	stimulus duration(ms)
Type 1	Matsuoka_etal_2003	guinea pig	NL96	none	37	-8	1
	Iribie_etal_2006	guinea pig	RWH99	dynamic	23	-4	2
Type 2	Shannon_etal_2004	rabbit	dynamic	dynamic	39	-15	4
	Grandi_etal_2010	human	dynamic	dynamic	39	-9.5	4
Type 3	Mahajan_etal_2008	rabbit	dynamic	instantaneous	26	-30	2
	Iyer_etal_2004	human	dynamic	instantaneous	67	-25	2
Type 4	Hund_etal_2004	dog	instantaneous	instantaneous	29	-30	2
	Faber_etal_2000	guinea pig	instantaneous	instantaneous	25	-25.5	2
	Livshitz_etal_2007	guinea pig	instantaneous	instantaneous	18	-15	2
	Ohara_etal_2011	human	instantaneous	instantaneous	41	-80	0.5
	Priebe_etal_1998	human	instantaneous	instantaneous	22	-30	2
Type 5	Fox_etal_2002	dog	none	instantaneous	13	-80	1
	TenTusscher_etal_2006	human	none	instantaneous	19	-51	1
	Fink_etal_2008	human	none	instantaneous	27	-24	2

**Table 3:** General information of 14 electrophysiological models including parameters of *CaTRPN* (*K*_*d*_, *K*_*on*_, *K*_*off*_), approximate time to reach quiescent states, beat number to reach priming steady states and ESI,PESI ranges.

Model Type	Model Name	*K*_*d*_ (mM)	*K*_*on*_ (mM^-1^ms^-1^)	*K*_*off*_ (ms^-1^)	Quiescent time	Prime beat No	ESI(ms)	PESI(ms)
Type 1	Matsuoka_etal_2003	7.69E-04	3.90E+01	3.00E-02	20min	500	250–1200	100–1200
	Iribie_etal_2006	function of F	8.00E+01	3.00E-01	10min	500	200–1200	100–1200
Type 2	Shannon_etal_2004	6.00E-04	3.27E+01	1.96E-02	20min	1500	200–1200	100–2000
	Grandi_etal_2010	6.00E-04	3.27E+01	1.96E-02	20min	500	300–1200	200–1200
Type 3	Mahajan_etal_2008	6.00E-04	3.27E+01	1.96E-02	10min	1500	300–1200	200–1200
	Iyer_etal_2004	1.00E-03	4.00E+01	4.00E-02	40min	15000	250–1500	200–1500
Type 4	Hund_etal_2004	5.00E-04	3.58E+01	1.79E-02	80min	6000~10500	250–1200	150–1200
	Faber_etal_2000	5.00E-04	3.58E+01	1.79E-02	20min	1500	200–1500	350–1500
	Livshitz_etal_2007	5.00E-04	3.58E+01	1.79E-02	20min	1500	200–1200	200–1200
	Ohara_etal_2011	5.00E-04	3.58E+01	1.79E-02	20min	1000	300–1200	200–1200
	Priebe_etal_1998	5.00E-04	3.58E+01	1.79E-02	20min	1500	400–2000	400–2000
Type 5	Fox_etal_2002	6.00E-04	3.27E+01	1.96E-02	20min	1500	200–2000	200–2000
	TenTusscher_etal_2006	1.00E-03	2.53E+01	2.50E-02	20min	1500	400–1000	450–1000
	Fink_etal_2008	1.00E-03	2.53E+01	2.50E-02	40min	1500	350–1200	350–1200

#### Type One: models with contraction

Two of the fourteen models have contraction in their original versions: Iribe_etal_2006[30] and Matsuoka_etal_2003[29].

Matsuoka_etal_2003 already has NL96 contraction in the original version. However, to investigate how much influence the mechanics has on this model, we remove the NL96 model and make *Ca* ^2+^ troponin (*CaTRPN*) a single-state-variable dynamic buffer (original model is a four-state variable dynamic buffer):

$$\frac{d[CaTRPN]}{dt}=K_{on}{\left[Ca^{2+}\right]}_{i}(B_{\text{max},troponin}-[CaTRPN])-K_{off}[CaTRPN]$$ (6)

[*Ca* ^2+^]_*i*_ is the intracellular *Ca* ^2+^ concentration; *B*_max,*troponin*_ is the total Troponin concentration; *K*_*on*_ and *K*_*off*_ are rate constants for the chemical reaction:

$$Ca+Troponin\overset{\;K_{on}\;}{\underset{\;K_{off}\;}{⇌}}CaTRPN$$ (7)

We set *K*_*on*_ and *K*_*off*_ to the values of *Y*1 and *Z*1 and *B*_max,*troponin*_ to the value in the original model. We call this new model Matuoska_etal_2003 without Contraction.

Iribe_etal_2006 includes the contraction model of Rice et al. (RWH99) in the original version [25]. For RWH99, *CaTRPN* is expressed by a single-state dynamic equation like Eq 6 but with a dynamic rate constant *K*_*off*_ that depends on force, hence it is strongly coupled even for isometric contractions. Also the RWH99 model has six tropomyosin/cross-bridge states with rate constants that are functions of the *Ca* ^2+^ troponin buffer. To simulate this model without the contraction part, we eliminated the six tropomyosin/cross-bridge states and set *K*_*off*_ for *CaTRPN* to a constant value by fixing the force in *K*_*off*_ expression to half of its maximum value. We then compare simulations of the original model and the version without contraction as well as one with NL96 contraction model (implemented as described in the next “Type Two” section).

#### Type Two: models with full dynamic buffers

Two of the models incorporate ordinary differential equations (ODEs) for all the buffers: Shannon_etal_2004[33] and Grandi_etal_2010[37]. The equations for the original *Ca* ^2+^ troponin buffers in these models are the same as Eq 6. For models of Type Two we add the four-state NL96 model using Eqs 2–5 above; this is done by splitting the dynamical *Ca* ^2+^ troponin buffer in the original model into two states: with (TCa*) or without (*TCa*) cross-bridges (Eqs 3 and 4):

$$\frac{d[CaTRPN]}{dt}=\frac{d[TCa]}{dt}+\frac{d[{TCa}^{*}]}{dt}$$ (8)

and adding the other two states *T* and T* (Eqs 2 and 5). There are multiple *K*_*on*_ and *K*_*off*_ in Eqs 2–4: *Yd*, *Y*1∼*Y*4, *Z*1∼*Z*3. They are kept the same as original NL96 paper[23] except for *Y*_1_ and *Z*_1_, which are chosen to match the values of *K*_*on*_ and *K*_*off*_ in Eq 6 from the original model (see Table 3). This is to preserve the dynamics of *CaTRPN* as similar as possible to the original model.

#### Type Three: models with dynamic *Ca* ^2+^ troponin buffers

Two of the models use ODEs for *CaTRPN* but instantaneous forms for all the other buffers: Mahajan_etal_2008[34] and Iyer_etal_2004[38].

For this type of models we follow the same step regarding the dynamic *Ca* ^2+^ troponin buffer in Type Two above and keep all the other buffers instantaneous. The only difference between Type Three and Type Two is that there are instantaneous buffer factors in Type Three models (see Eqs 9 and 10 below) but not in Type two.

#### Type Four: models with instantaneous *Ca* ^2+^ troponin buffers

Five of the models have all instantaneous *Ca* ^2+^ buffers: Hund_etal_2004[35]; Faber_etal_2000[31]; Livshitz_etal_2007[32]; Ohara_etal_2011[39] and Priebe_etal_1998[40]. The buffering of *Ca* ^2+^ by Troponin is represented using the following equations:

$$\frac{d{\left[Ca^{2+}\right]}_{i}}{dt}=\beta I_{total}^{Ca}$$ (9)

$$\beta =1/(1+\sum_{j}\frac{B_{\text{max},j}K_{d,j}}{{\left({\left[Ca^{2+}\right]}_{i}+K_{d,j}\right)}^{2}}),\;j:\;\text{all intracellular}\;{Ca}^{2+}\;\text{buffers}$$ (10)

$I_{total}^{Ca}$ represents the total intracellular *Ca* ^2+^ flux (see Page 18 in the supplement in reference [39] for detailed fomula); *β* is the instantaneous buffer factor; index *j* represents each type of intracellular *Ca* ^2+^ buffer; *B*_max, *j*_ and *K*_*d*,*j*_ are the total concentration and the affinity constant for buffer *j*. For this type of model we first change the instantaneous intracellular *Ca* ^2+^ troponin buffer into a dynamic buffer by eliminating the *CaTRPN* term from the instantaneous buffer factor *β* and by adding a dynamic *CaTRPN* flux into the [*Ca* ^2+^]_*i*_ equation:

$$\frac{d{\left[Ca^{2+}\right]}_{i}}{dt}=\beta '(I_{total}^{Ca}-I_{trop})$$ (11)

$$\beta '=1/(1+\sum_{j}\frac{B_{\text{max},j}K_{d,j}}{{\left({\left[Ca^{2+}\right]}_{i}+K_{d,j}\right)}^{2}}),j:\;\text{all}\;\text{intracellular}\;{Ca}^{2+}\;\text{buffers except for}\;CaTRPN$$ (12)

$$I_{trop}=\frac{d[CaTRPN]}{dt}=K_{on}{\left[Ca\right]}_{i}(B_{\text{max},troponin}-[CaTRPN])-K_{off}[CaTRPN]$$ (13)

The way to choose *K*_*on*_ and *K*_*off*_ here is not unique as long as $\frac{K_{off}}{K_{on}}=K_{d}$ in the original model. We choose $K_{off}=\sqrt{\sigma }K_{off,s}\;{\text{K}}_{\text{off}}=\sqrt{\sigma }{\text{K}}_{\text{offs}},{\text{K}}_{\text{on}}=\frac{1}{\sqrt{\sigma }}{\text{K}}_{\text{on},\text{s}}$ and $K_{on}=\frac{1}{\sqrt{\sigma }}K_{on,s}$, where *s* indicates the values from the Shannon_etal_2004 model and $\sigma =\frac{K_{d}}{K_{d,s}}$. We use the values from Shannon_etal_2004 as the standard values here because they have been widely used by to simulate dynamic *CaTRPN* buffers. After changing the instantaneous *CaTRPN* into a dynamical buffer, we follow the same procedure as for Type Three models.

#### Type Five: models without *Ca* ^2+^ troponin buffers

Three of the models do not have *Ca* ^2+^ troponin buffers, but they do have other intracellular *Ca* ^2+^ buffers: Fox_etal_2002[36]; TenTusscher_etal_2006[41] and Fink_etal_2008[42].

For this type of models, we first add instantaneous *Ca* ^2+^ troponin buffers into the models. If the model has one general *Ca* ^2+^ buffer (referred as General) representing the average effect of all intracellular *Ca* ^2+^ buffers (e.g. TenTusscher_etal_2006 and Fink_etal_2008), we split the general buffer into two parts: *CaTRPN* and “Other”. We keep *K*_*d*,*TRPN*_ and *K*_*d*,*Other*_ to be the same as *K*_*d*,*General*_ in the original model so that the *Ca* ^2+^ affinity of the instantaneous buffer will be retained. [*Troponin*]_*total*_ was set to be 0.07mM, which is a standard value in most models. [*Other*]_*total*_ = [*General*]_*total*_ − [*Troponin*]_*total*_ so that the concentration of the total intracellular *Ca* ^2+^ buffer is the same as in the original model. If the model has other *Ca* ^2+^ buffers (such as CMDN) but no *CaTRPN* (e.g. Fox_etal_2002), we keep the other buffers unchanged and add a *CaTRPN* buffer. For the new *CaTRPN* buffer we also set [*Troponin*]_*total*_ = 0.07*mM* and *K*_*d*,*CaTRPN*_ = 0.6*μM* which are both common values in many models. After adding an instantaneous *CaTRPN* to the model, we follow the steps for Type Four to implement NL96.

### Numerical integration

Except for one model (Iyer_etal_2004), all the code for the original single cell models were downloaded from www.cellml.org in CELLML format. Then they were translated into.mat files (MATLAB files) using a PYCML program.[43][44] Simulations were run in MATLAB and integrated using the forward Euler integration method with time steps of *dt* = 0.001*ms*. The Iyer_etal_2004 model consists of 67 variables and requires a *dt* integration step as low as 1×10^−5^ *ms* to converge[38] using forward Euler, thus becoming impractically slow to simulate in MATLAB. Therefore as in [45] the Iyer_etal_2004 model was written in FORTRAN using a semi-implicit integration method that allows a much larger (while still convergent) integration time step of *dt* = 0.005*ms*.

### Pacing protocol and initial conditions

Our pacing protocol is composed of three steps and similar to the pacing protocol from Yue et al [22] except that we chose to use a priming period of 500ms. The stimulus currents and durations for the different models were selected to ensure excitation and the values are provided in Table 2. All simulations were run for isometric contraction of a single cell, where the half sarcomere length is fixed to be 1.05μm.

#### Step one: Quiescent

In this step, EP models without contractions were run with no stimulation current until quiescent steady states were reached (i.e., no state variable changed by more than 0.01%). Initial values for state variables in each model were directly loaded from CellML code. The time for each model to reach the quiescent state is listed in Table 3, and it ranges from 10min of real time (as in the Mahajan_etal_2008 model) to up to 100min (as in the Hund_etal_2004 model). Note that these are real times and not run times. Steady state quiescent values for voltage, intracellular and JSR (or SR) *Ca* ^2+^ concentrations vary considerably among models as shown in Table 4.

**Table 4:** Quiescent state variables: membrane voltage, intracellular calcium condensation and JSR (or SR) calcium condensation.

Model Type	Model name	whether original*	Vm (mV)	[Ca^2+^]_i_ (mM)	[Ca^2+^]JSR (mM)	[Ca^2+^]SR(mM)
Type 1	Matsuoka_etal_2003	No	-85.9	2.92E-06	4.76E+00	5.50E+00
	Iribie_etal_2006	No	-94.3	5.50E-06	NA	6.82E-03
Type 2	Shannon_etal_2004	Yes	-85.4	7.06E-05	NA	4.58E-01
	Grandi_etal_2010	Yes	-81.0	7.08E-05	NA	4.60E-01
Type 3	Mahajan_etal_2008	Yes	-86.4	5.31E-05	3.68E-02	7.36E-02
	Iyer_etal_2004	Yes	-91.6	1.40E-05	3.92E-02	7.84E-02
Type 4	Hund_etal_2004	Yes	-86.9	8.34E-05	1.27E+00	2.54E+00
	Faber_etal_2000	Yes	-85.3	7.19E-05	1.09E+00	2.18E+00
	Livshitz_etal_2007	Yes	-90.0	8.00E-05	7.20E+00	8.40E+00
	Ohara_etal_2011	Yes	-88.3	5.58E-05	9.82E-01	1.96E+00
	Priebe_etal_1998	Yes	-91.6	9.26E-05	1.58E+00	3.17E+00
Type 5	Fox_etal_2002	Yes	-94.3	5.02E-05	NA	1.11E+00
	TenTusscher_etal_2006	Yes	-87.5	2.58E-05	NA	1.87E-01
	Fink_etal_2008	Yes	-86.8	9.05E-05	NA	2.05E+00

* If the original model does not have contraction, we run the original model to get quiescent state. If the original model has contraction, we take out the contraction part and run that to quiescent state.

#### Step Two: Priming Cycle

During the priming pacing cycle, all models (with and without contractions) were paced with priming period T = 500ms starting from the quiescent states at the end of Step one, until a new “priming” steady state was achieved. The beat number required to reach this priming steady state for each model is listed in Table 2. For models incorporating NL96 contraction, state variables for the four states of *Ca* ^2+^ troponin buffer were calculated by solving Eqs 2–5 for steady state using the quiescent value of [*Ca* ^2+^]_*i*_. The total *Ca* ^2+^ troponin buffer ([*TCa*]+[TCa*]) calculated using this method was verified to be similar to the quiescent value in the original EP model if the original model had a *Ca* ^2+^ troponin buffer. We used this method to ensure that the initial conditions for the original models and the corresponding contraction models were as close as possible.

#### Step Three: Postextrasystolic pacing protocol

For all models, an extrasystolic (ES) and a postextrasystolic (PES) beat were delivered in sequence after the last priming beat as described in the Contraction Model and Experiment section. We held ESI at a constant value and varied PESI to record the transients of transmembrane potential, [*Ca* ^2+^]_*i*_, *CaTRPN* and generated force; we then changed ESI to a different value and repeated the process using the priming steady state as initial conditions. The shortest PESI is the refractory period of the ES beat so it is just long enough that the PES beat is separated from the ES beat. The longest PESI (ESI) value was chosen as 1200ms, 1500ms or 2000ms, depending on the time it took for the Postextrasystolic *MRC*_*pes*_ (*MRC*_*es*_) to converge to a plateau level, as some models took longer than others to reach it.

### Data Analysis

All curves were fitted using MATLAB’s built-in function “fit” whose default curve fit algorithm is the Trust-Region method. The value of r^2^ was given for each fit and 95% confidence bounds were provided for each fitted coefficient. Our analysis is divided into two parts: **Ca** ^**2+**^ **dynamics** and **Contraction.** To analyze **Ca** ^**2+**^ **dynamics** we compare the *Ca* ^2+^ transients for the priming beats and during postextrasystolic potentiation between the original models and models with contraction. To analyze **Contraction** we generate four characteristic contraction curves and fit two of them into monoexponential functions.

#### Ca^2+^ Dynamics

**Priming** *Ca* ^2+^: For each model, we plotted the intracellular *Ca* ^2+^ of the last priming beat, i.e. priming steady state, for both the original model and the one with contraction implemented in the same plot. We quantified the *Ca* ^2+^ transient by calculating the following: 1) minimum (diastolic) [*Ca* ^2+^]_*i*_; 2) maximum (systolic) [*Ca* ^2+^]_*i*_; 3) the duration for which [*Ca* ^2+^]_*i*_ was above its half amplitude level, i.e. [*Ca* ^2+^]_*i*_ ≥ [*Ca* ^2+^]_*diastolic*_ + ([*Ca* ^2+^]_*systolic*_ − [*Ca* ^2+^]_*diastolic*_) / 2; 4) the time at which [*Ca* ^2+^]_*i*_ reached its maximum systolic value (*t*_*peak*_); and 5) the amplitude of the total *Ca* ^2+^ charge delivered to the cytoplasmic compartment during one beat (*Q*) that was calculated by integrating the area underneath the [*Ca* ^2+^]_*i*_ − [*Ca* ^2+^]_*diastolic*_ transient. We quantified the differences of the parameters between original models and models with contractions by the relative difference, defined as

$$\Delta Parameter=(Model_{WithNL96Contraction}-Model_{WithoutContraction})/Model_{WithoutContraction}$$ (14)

**Postextrasystolic Potentiation in [*Ca*^2+^]_*i*_**: For each model, we plotted the intracellular *Ca* ^2+^ transient for the last 3 priming beats, along with multiple ES/PES beat pairs overlaid on the same graph for each model (without contraction on the left and with contraction of the right).

#### Contraction

Following the data fitting and analysis from Yue paper[22] with modification, we generate four characteristic curves for each model after the implementation of contraction: **Postextrasystolic Mechanical Restitution Curve (*MRC***_***pes***_ **), Postextrasystolic Potentiation Curve (PESPC), Minimum-value Axis Intercept Curve (*t***_***o*,*pes***_ **plotted vs ESI) and Time Constant Curve (*T***_***mrc*,*pes***_ **plotted vs ESI).**

**Postextrasystolic (*PES*) Mechanical Restitution Curves (*MRC***_***pes***_ **)** are a family of curves of maximum rate of change of force of postextrasystolic beats normalized to the last priming beat plotted vs PESI. *For a fixed ESI*, *MRC*_*pes*_ increases monoexponentially to a plateau level (the fully restituted value) as PESI increases. The equation for this *MRC*_*pes*_ is:

$$\frac{dF\;/\;dt_{\text{max}}(PES)}{dF\;/\;dt_{\text{max}}(SS)}=CR_{\text{max},pes}\{1-\text{exp}[-\frac{PESI-t_{o,pes}}{T_{mrc,pes}}]\}+C_{0}$$ (15)

where *F* denotes the force generated from NL96 model; *PES* denotes that it is the postextrasystolic beat; *SS* denotes the steady state during the priming period; *CR*_max,*pes*_ is the plateau amplitude, termed as Maximum Postextrasystolic Contractile Response; *C*_0_ is the minimum value of $\frac{dF\;/\;dt_{\text{max}}(PES)}{dF\;/\;dt_{\text{max}}(SS)}$, in Yue et al.[22] *C*_0_ = 0; *t*_*o*,*pes*_ is the minimum-value axis intercept where *MRC*_*pes*_ intercepts the minimum value line $\frac{dF\;/\;dt_{\text{max}}(PES)}{dF\;/\;dt_{\text{max}}(SS)}=C_{0}$; and *T*_*mrc*,*pes*_ is the time constant for *MRC*_*pes*_.. Fig 1(A) shows one set of *MRC*_*pes*_ generated using equations and parameters in Yue et al.[22].

Extrasystolic (*PES*) Mechanical Restitution Curves (*MRC*_*es*_) presents the maximum rates of change of force of extrasystolic beats normalized to the last priming beat (*dF* / *dt*_max_ (*ES*) / *dF* / *dt*_max_(*SS*)) as a function of ESI. Since it is a subset of *MRC*_*pes*_ with ESI equal to the priming cycle length, we do not present them separately.

**Postextrasystolic Potentiation Curve (PESPC)** is the maximum postextrasystolic contractile response (*CR*_max,*pes*_) plotted vs ESI. *CR*_max,*pes*_ is the plateau amplitude of the above *MRC*_*pes*_ for a fixed ESI and this amplitude decreases to a plateau value as ESI increases. Fig 1(B) shows the PESPC and the simultaneously determined *MRC*_*es*_ **,** both generated from the equations in Yue et al.[22]. The equation for PESPC fitting is:

$$CR_{\text{max},pes}=B+A\{\text{exp}[\frac{ESI-t_{o,es}}{T_{pespc}}]\}$$ (16)

where *B* is the plateau level; *A* is the amplitude; *t*_*o*,*es*_ is the ESI-axis intercept for the simultaneously determined Extrasystolic Mechanical Restitution Curve; *T*_*pespc*_ is the time constant for PESPC.

## Results

Our Results section is divided into three parts: **Calcium Results, Contraction Results,** and the **Underlying Mechanism for PESP**. In the **Calcium Results** section we first compared the **Priming** *Ca* ^2+^ transients between original EP models and the corresponding models with the NL96 contraction model to investigate the influence of including contraction on EP models. Then we studied the **Postextrasystolic Potentiation behavior in** *Ca* ^2+^ before and after the implementation of contraction. In the **Contraction Results** section we compared the four characteristic contractile curves with the Yue et al. experimental results. In the **Underlying Mechanism for PESP** section we analyzed the mechanism of PESP by finding the correlation between the calcium release from SR and the contractile strength.

### Ca^2+^ Results

#### Priming [*Ca* ^2+^]_*i*_ properties are undisturbed after the implementation of contraction for models with dynamic buffers

We present the priming [*Ca* ^2+^]_*i*_ data in Fig 2, Tables 5, 6, 7 and the S2 File. Fig 2(A)–2(F) illustrates [*Ca* ^2+^]_*i*_ transients for the last priming beat for six representative models with (dashed lines) and without (solid lines) contraction. They are chosen to include five of the different EP types: (a) Matsuoka_etal_2003 (Type One: with contraction(NL96)); (b) Iribe_etal_2006 (Type One: with contraction(RWH99)); (c) Shannon_etal_2004 (Type Two: full dynamic *Ca* ^2+^ buffer); (d) Mahajan_etal_2008 (Type Three: dynamic *Ca* ^2+^ troponin buffer); (e) Ohara_etal_2004 (Type Four: instantaneous *Ca* ^2+^ troponin buffer); (f) TenTusscher_etal_2006 (Type Five: no *Ca* ^2+^ troponin buffer). The corresponding plots for all the fourteen models are provided in S2 File.The five quantities computed to quantify the priming [*Ca* ^2+^]_*i*_ transient for all EP models without (with) contraction are provided in in Table 5 (Table 6) and the relative differences between before and after contraction are shown in Table 7, where double dagger symbols (‡) indicate the differences larger than 0.5(50%); single dagger symbols (†) indicate differences larger than 0.1 (10%) but less than 0.5 (50%).

**Table 5:** Data for steady priming beat: EP models without contraction.

Model Type	Model Name	[Ca^2+^]i_diastolic(mM)	[Ca^2+^]i_systolic(mM)	t1/2(ms)	tpeak(ms)	Q (mM*ms)
Type 1	Matsuoka_etal_2003	1.46E-05	1.50E-03	5.69E+01	2.02E+01	9.80E-02
	Iribie_etal_2006	1.03E-05	1.50E-03	5.25E+01	2.08E+01	8.40E-02
Type 2	Shannon_etal_2004	1.13E-04	4.80E-04	1.41E+02	4.60E+01	5.90E-02
	Grandi_etal_2010	1.11E-04	3.82E-04	1.42E+02	5.49E+01	4.49E-02
Type 3	Mahajan_etal_2008	2.04E-04	5.61E-04	1.94E+02	7.90E+01	7.57E-02
	Iyer_etal_2004	2.33E-04	9.90E-04	2.23E+02	7.80E+01	1.80E-01
Type 4	Hund_etal_2004	1.86E-04	7.37E-04	1.64E+02	4.27E+01	1.02E-01
	Faber_etal_2000	2.45E-04	2.00E-03	9.75E+01	1.47E+01	2.04E-01
	Livshitz_etal_2007	1.73E-04	1.40E-03	1.20E+02	2.20E+01	1.61E-01
	Ohara_etal_2011	1.18E-04	6.52E-04	1.37E+02	4.31E+01	8.74E-02
	Priebe_etal_1998	4.31E-04	9.73E-04	1.75E+02	1.52E+01	1.10E-01
Type 5	Fox_etal_2002	3.80E-05	1.62E-03	8.22E+01	1.75E+01	1.52E-01
	TenTusscher_etal_2006	1.51E-04	1.50E-03	1.38E+02	2.93E+01	2.10E-01
	Fink_etal_2008	1.42E-04	1.50E-03	1.02E+02	3.43E+01	1.72E-01

**Table 6:** Data for steady priming beat: models with NL96 contraction.

Model Type	Model Name	[Ca^2+^]i_diastolic(mM)	[Ca^2+^]i_systolic(mM)	t1/2 (ms)	tpeak (ms)	Q (mM*ms)
Type 1	Matsuoka_etal_2003	1.49E-05	1.60E-03	5.65E+01	2.01E+01	1.02E-01
	Iribie_etal_2006	1.04E-05	1.40E-03	5.26E+01	2.06E+01	8.19E-02
Type 2	Shannon_etal_2004	1.12E-04	4.75E-04	1.46E+02	5.80E+01	5.99E-02
	Grandi_etal_2010	1.14E-04	3.87E-04	1.35E+02	5.48E+01	4.38E-02
Type 3	Mahajan_etal_2008	2.06E-04	5.70E-04	1.89E+02	7.80E+01	7.49E-02
	Iyer_etal_2004	2.41E-04	1.00E-03	2.16E+02	7.60E+01	1.83E-01
Type 4	Hund_etal_2004	1.95E-04	1.20E-03	7.05E+01	1.18E+01	1.06E-01
	Faber_etal_2000	2.27E-04	4.80E-03	6.90E+00	3.79E+00	1.80E-01
	Livshitz_etal_2007	1.86E-04	2.30E-03	2.99E+01	1.60E+01	1.19E-01
	Ohara_etal_2011	1.29E-04	1.00E-03	5.81E+01	1.66E+01	9.00E-02
	Priebe_etal_1998	4.23E-04	1.60E-03	1.93E+01	1.01E+01	1.11E-01
Type 5	Fox_etal_2002	1.16E-04	2.79E-04	1.86E+02	1.38E+01	3.35E-02
	TenTusscher_etal_2006	1.59E-04	2.00E-03	7.47E+01	2.59E+01	2.15E-01
	Fink_etal_2008	1.49E-04	2.00E-03	5.99E+01	3.14E+01	1.75E-01

**Table 7:** Data for steady priming beat: relative differences between EP models and models with NL96 contractions.

Model Type	Model Name	Δ[Ca^2+^]i_diastolic	Δ[Ca^2+^]i_systolic	Δt1/2	Δtpeak	ΔQ
Type 1	Matsuoka_etal_2003	1.80E-02	6.67E-02	-7.33E-03	-7.52E-03	3.78E-02
	Iribie_etal_2006	1.06E-02	-6.67E-02	1.54E-03	-9.41E-03	-2.55E-02
Type 2	Shannon_etal_2004	-5.31E-03	-1.02E-02	3.55E-02	2.61E-01‡	1.53E-02
	Grandi_etal_2010	2.23E-02	1.23E-02	-4.68E-02	-2.31E-03	-2.45E-02
Type 3	Mahajan_etal_2008	1.03E-02	1.60E-02	-2.58E-02	-1.27E-02	-9.66E-03
	Iyer_etal_2004	3.31E-02	1.03E-02	-3.15E-02	-2.56E-02	1.72E-02
Type 4	Hund_etal_2004	4.82E-02	6.28E-01‡	-5.71E-01‡	-7.25E-01‡	3.53E-02
	Faber_etal_2000	-7.55E-02	1.60E+00‡	-9.29E-01‡	-7.43E-01‡	-1.21E-01†
	Livshitz_etal_2007	7.47E-02	6.43E-01‡	-7.51E-01‡	-2.73E-01†	-2.61E-01†
	Ohara_etal_2011	9.24E-02	5.34E-01‡	-5.75E-01‡	-6.15E-01‡	2.97E-02
	Priebe_etal_1998	-1.69E-02	6.44E-01‡	-8.90E-01‡	-3.36E-01†	1.46E-02
Type 5	Fox_etal_2002	2.05E+00‡	-8.28E-01‡	1.26E+00‡	-2.12E-01†	-7.79E-01‡
	TenTusscher_etal_2006	5.20E-02	3.33E-01†	-4.59E-01†	-1.15E-01†	2.43E-02
	Fink_etal_2008	4.86E-02	3.33E-01†	-4.11E-01†	-8.53E-02	2.10E-02

† The relative difference is between 10% and 50%

‡ The relative difference is more than 50%

[*Ca* ^2+^]_*i*_ differs substantially among the original EP models (without contraction) as shown in Table 5. For example, for systolic [*Ca* ^2+^]_*i*_, Grandi_etal_2010 and Shannon_etal_2004 have values in the [0.30,0.50]×10^−3^ *mM* range while Faber_etal_2000 is up to four times larger at 2.00×10^−3^ *mM*; for diastolic [*Ca* ^2+^]_*i*_, the maximum value is 2.00×10^−3^ *mM* for Faber_etal_2000 and the minimum value is 3.82×10^−4^ *mM* for Grandi_etal_2010, which is one fifth of the maximum value.

The original models that include dynamic *Ca* ^2+^ troponin buffers (Type One: Fig 2 panels (a) and (b); Type Two: panel (c); and Type Three: panel (d)) do not exhibit a significant change in the shape of the priming [*Ca* ^2+^]_*i*_ transient after implementing contraction (adding NL96 model), but the models with instantaneous, or no *Ca* ^2+^ troponin buffers (Type Four: panel (e); and Type Five: panel (f)), demonstrate large differences in the [*Ca* ^2+^]_*i*_ transient shape.

**Fig 3:**
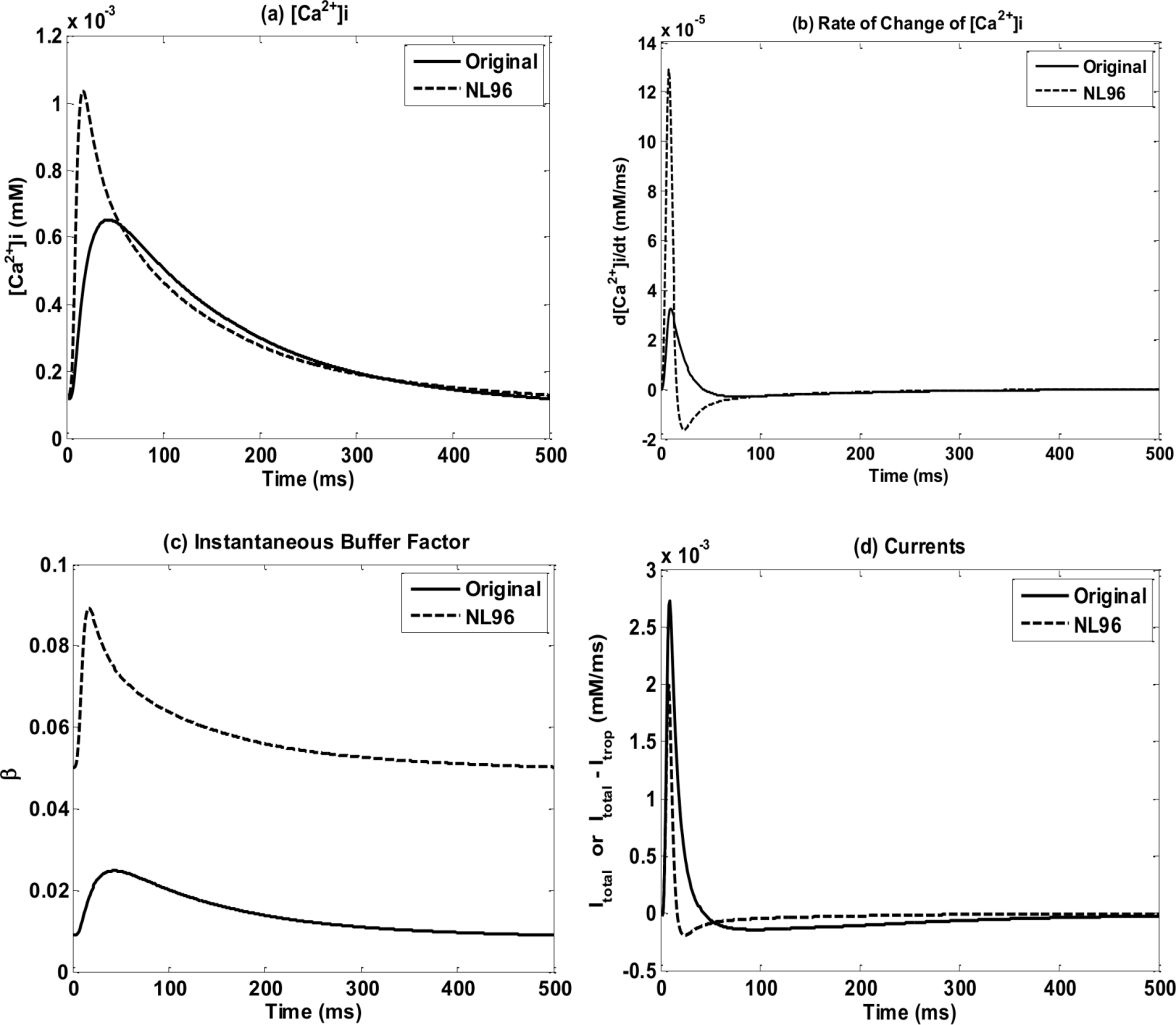
*Ca* ^2+^ shape changes for models with instantaneous buffers. Figures show the O’hara_etal_2008 model for: (a) [*Ca* ^2+^]_*i*_ (grey lines) and *d*[*Ca* ^2+^]_*i*_ / *dt* (black lines), before (solid lines) and after (dash lines) the implementation of NL96; (b) zoom in of *d*[*Ca* ^2+^]_*i*_ / *dt*; (c) the instantaneous buffer factor *β* before (solid line) and after (dash line) the implementation of NL96; (d) intracellular *Ca* ^2+^ flux before (solid line) and after (dash line) the implantation of NL96.

The most significant change tended to be an increase in the maximum [*Ca* ^2+^]_*i*_, always accompanied by a decrease in the time of this peak (*t*_*peak*_). As shown in Table 7, all six models with dynamic *Ca* ^2+^ troponin buffers have relative differences of less than 10% in systolic [*Ca* ^2+^]_*i*_ and five out them have relative differences less than 10% in *t*_*peak*_. On the other hand, among the eight models with instantaneous or none *Ca* ^2+^ troponin buffers, six models show differences larger than 50% and none has a difference less than 10% in systolic [*Ca* ^2+^]_*i*_; in *t*_*peak*_, one of the eight models (Fink_etal_2008) has a relative difference less than 10% and three of them have differences of more than 50%. In addition, except for one model (Fox_etal_2002), all of them have positive relative differences in systolic [*Ca* ^2+^]_*i*,_ indicating a systolic [*Ca* ^2+^]_*i*_ increase after the implementation of the NL96 contraction; and all eight models have negative Δ*t*_*peak*_, meaning the time of the peak is decreased by the implementation of contraction. The increase in maximum [*Ca* ^2+^]_*i*_ and the decrease in *t*_*peak*_ in models with instantaneous *Ca* ^2+^ troponin buffers after the implementation of NL96 (Fig 2, S2 File) is due to an increased and fast rate change of the intracellular calcium after its dynamics has been modified from Eqs 9 and 10 (before the implementation) to Eqs 11 and 12 (after the implementation) to account for the contraction. Fig 3(A) and 3(B) shows, as an example, the [*Ca* ^2+^]_*i*_ and *d*[*Ca* ^2+^]_*i*_ / *dt* respectively for the Ohara_etal_2011 model before (solid lines) and after (dashed line) the NL96 implementation. It can be seen that after the implementation of NL96: *(i)* the instantaneous buffer factor *β* increases due to the elimination of the instantaneous *Ca* ^2+^ troponin buffer from the denominator (see Eqs 10 and 12); and (ii) the flux $I_{total}^{Ca}$ is reduced due to the addition of the negative dynamic *Ca* ^2+^ troponin flux (see Eqs 9 and 11). The flux $I_{total}^{Ca}$ decreases by less than 50% of the original value (Fig 3(D)) but *β* increases by more than 200% (Fig 3(C)) resulting in an sharp increase in [*Ca* ^2+^]_*i*_ amplitude. And the time for *β* to reach its peak is clearly shortened, resulting in the decrease of [*Ca* ^2+^]_*i*_ peak (*t*_*peak*_).

Results for the priming [*Ca* ^2+^]_*i*_ duration (*t*_1/2_) are similar to those of systolic [*Ca* ^2+^]_*i*_ (see Table 7). All six models that have dynamic *Ca* ^2+^ troponin buffers show little change in *t*_1/2_ (less than 10%). Among the eight models with instantaneous or no *Ca* ^2+^ troponin buffers, six have differences more than 50% and none has a difference less than 10%. Seven out of eight have negative Δ*t*_1/2_, indicating the shortening in the time duration when [*Ca* ^2+^]_*i*_ stays above half the amplitude. This large difference in *t*_1/2_ is closely related to the change in systolic [*Ca* ^2+^]_*i*_. A sharp increase in systolic [*Ca* ^2+^]_*i*_ greatly raises the half amplitude level (see the definition of the half amplitude level in Method section); this together with the large and narrow spike in [*Ca* ^2+^]_*i*_ generated by the inclusion of contraction, significantly decrease the time duration where [*Ca* ^2+^]_*i*_ stay above the half amplitude level.

The diastolic [*Ca* ^2+^]_*i*_ and the *Ca* ^2+^ charge (*Q*) during one beat are the two parameters that do not change much after implementing NL96 contraction in all of the models. Thirteen out of fourteen models have diastolic [*Ca* ^2+^]_*i*_ changes of less than 10%, with only one model having a difference larger than 50% (Fox_etal_2002). *Q* for eleven out of fourteen models changes by less than 10% and only one model (Fox_etal_2002) has a change of more than 50% (see Table 7).This is because, although the systolic [*Ca* ^2+^]_*i*_ is greatly increased for models with instantaneous or none *Ca* ^2+^ troponin buffers, its increase is localized to a relatively narrow spike with an area that is negligible comparing with that of the rest of the [*Ca* ^2+^]_*i*_ transient.

#### Models with dynamic buffers show significant Postextrasystolic Potentiation in [*Ca* ^2+^]_*i*_

**Fig 4:**
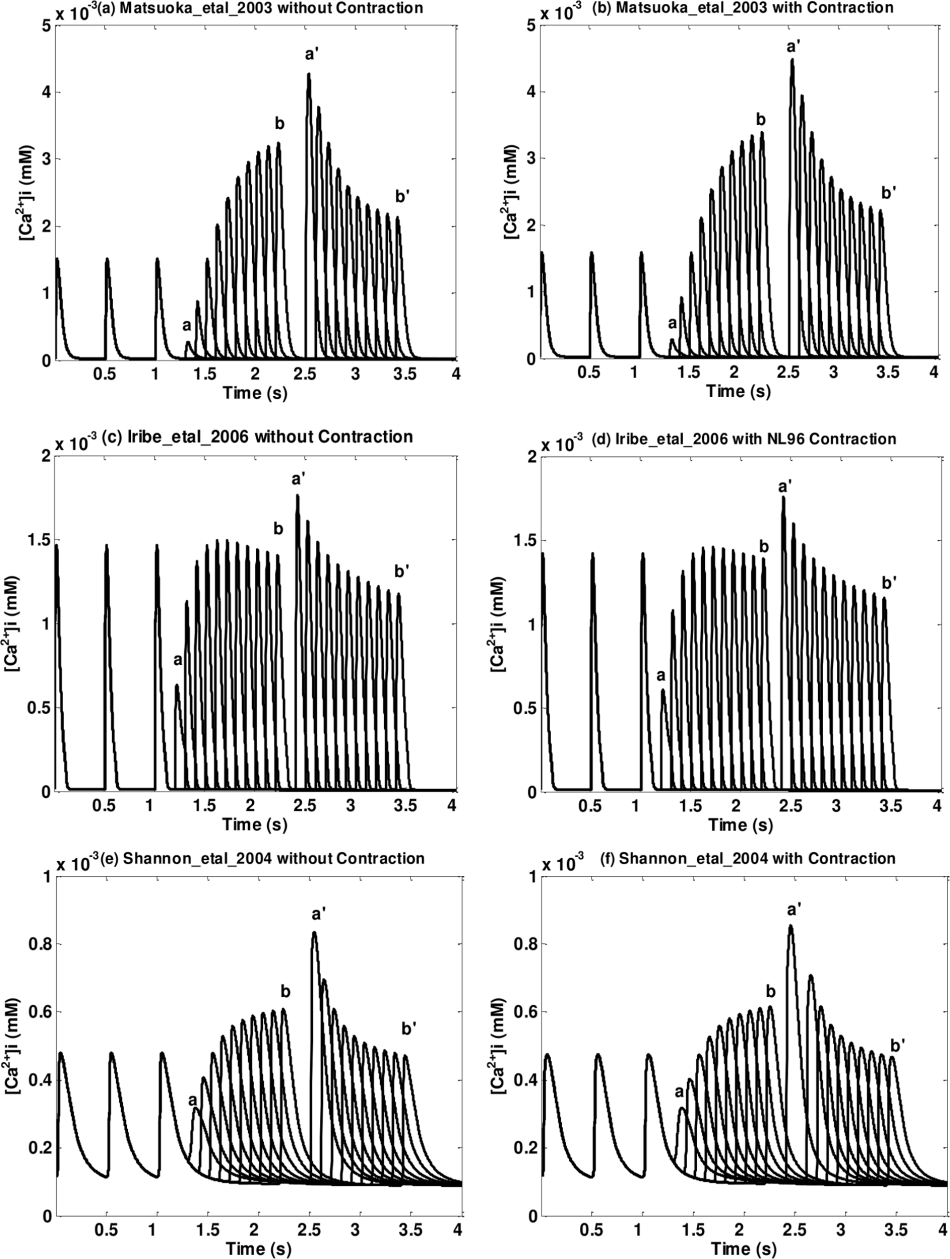
Postextrasystolic potentiation in [*Ca* ^2+^]_*i*_ transient for three models of Type One and Two. (a) (b) Matsuoka_etal_2003 without/with contraction; (c) (d) Iribe_etal_2006 without/with contraction (NL96); (e) (f) Shannon_etal_2004 without/with contraction. The first three beats are priming beats; from beat *a* to beat *b* are ES beats with different ESIs; from *a*' to *b*' are PES beats with a fixed PESI (fully restituted PESI, defined in Step Three in the Pacing Protocol of the Methods section); *a* and *a*' are corresponding ES and PES beats; so are *b* and *b*'. Note that each pair of figures (without/with contraction) has the same axis range.

**Fig 5:**
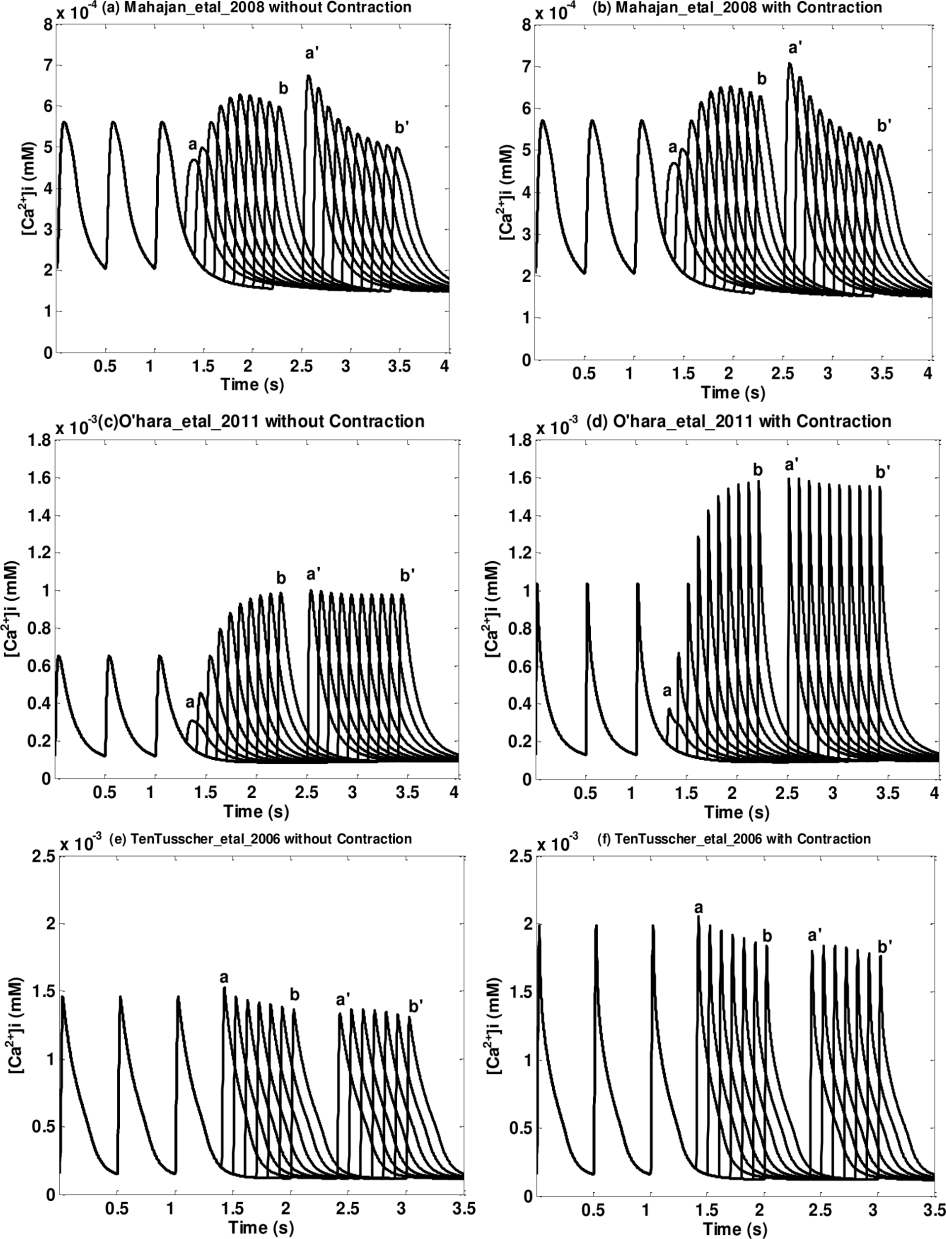
Postextrasystolic potentiation in [*Ca* ^2+^]_*i*_ transient for three models of Type Three, Four and Five. (a) (b) Mahajan_etal_2008 without/with contraction; (c) (d) O’hara_etal_2006 without/with contraction; (e) (f) TenTusscher_etal_2004 without/with contraction. As in the previous figure, the first three beats are priming beats; from beat *a* to beat *b* are ES beats with different ESIs; from *a*' to *b*' are PES beats with a fixed PESI And each pair of figures (without/with contraction) has the same axis range.

Postextrasystolic potentiation exists in intracellular *Ca* ^2+^ transients, where giving a fixed and long enough PESI, as the ESI increases, the amplitudes of the [*Ca* ^2+^]_*i*_ of the ES beats will increase while the PES beats will show the opposite trend. Representative examples of the differences in [*Ca* ^2+^]_*i*_ postextrasystolic potentiation before and after the implementation of NL96 contraction of Type One and Two (Type Three, Four, and Five) models are shown in Fig 4 (Fig 5). Figures for all models are in S3 File. ES beats with different ESIs are labeled from *a* to *b*; PES beats with a fixed PESI are labeled from *a*' to *b*' (see Step Three in Pacing Protocol in the Methods section); *a* and *a*' (*b* and *b*') correspond to ES and PES paired beats for the shortest (longest) ESI.

From Figs 4 and 5 and S3 File we can see that models with dynamic *Ca* ^2+^ troponin buffers show a significant postextrasystolic potentiation in the [*Ca* ^2+^]_*i*_ transients. As ESI increases, the amplitudes of the ES beats increase while the corresponding PES beats decrease. In addition, there’s no notable difference in [*Ca* ^2+^]_*i*_ transient before and after the implementation of NL96 contraction (see Fig 4 and S3 File). On the other hand, models with instantaneous *Ca* ^2+^ troponin buffers do not show a significant postextrasystolic potentiation in the [*Ca* ^2+^]_*i*_ transients. Among the eight models, only one model (Livshitz_etal_2007, see S3 File) shows notable amplitude change in both ES beats and PES beats. Three models (Hund_etal_2004, Faber_etal_2000 and Ohara_etal_2011, see Fig 5 and S3 File) show increase in ES beats but no noticeable decrease in PES beats. Four models (Priebe_etal_1998, Fox_etal_2002, TenTusscher_etal_2006 and Fink_etal_2008, see Fig 5 and S3 File) show neither considerable increase in ES beats nor decrease in PES beats. Note that these types of models, as discussed before, show a change in the shape of the [*Ca* ^2+^]_*i*_ when contraction is included. However it doesn’t change the postextrasystolic potentiation behavior much, namely, if the original model has a clear PESP trend, after the implementation, the new model will still maintain that behavior and vice versa.

### Contraction Results

To investigate how well our EP models with updated contraction can represent postextrasystolic dynamics, we calculated the four characteristic curves, described in the Methods, for all fourteen models with the NL96 contraction and compared them with the experimental data from Yue et al.[22]. Note that we did not show any CaTRPN transient because they are linearly related to the contraction in isometric condition[23].

#### Postextrasystolic Mechanical Restitution Curve (*MRC*_*pes*_)

**Fig 6:**
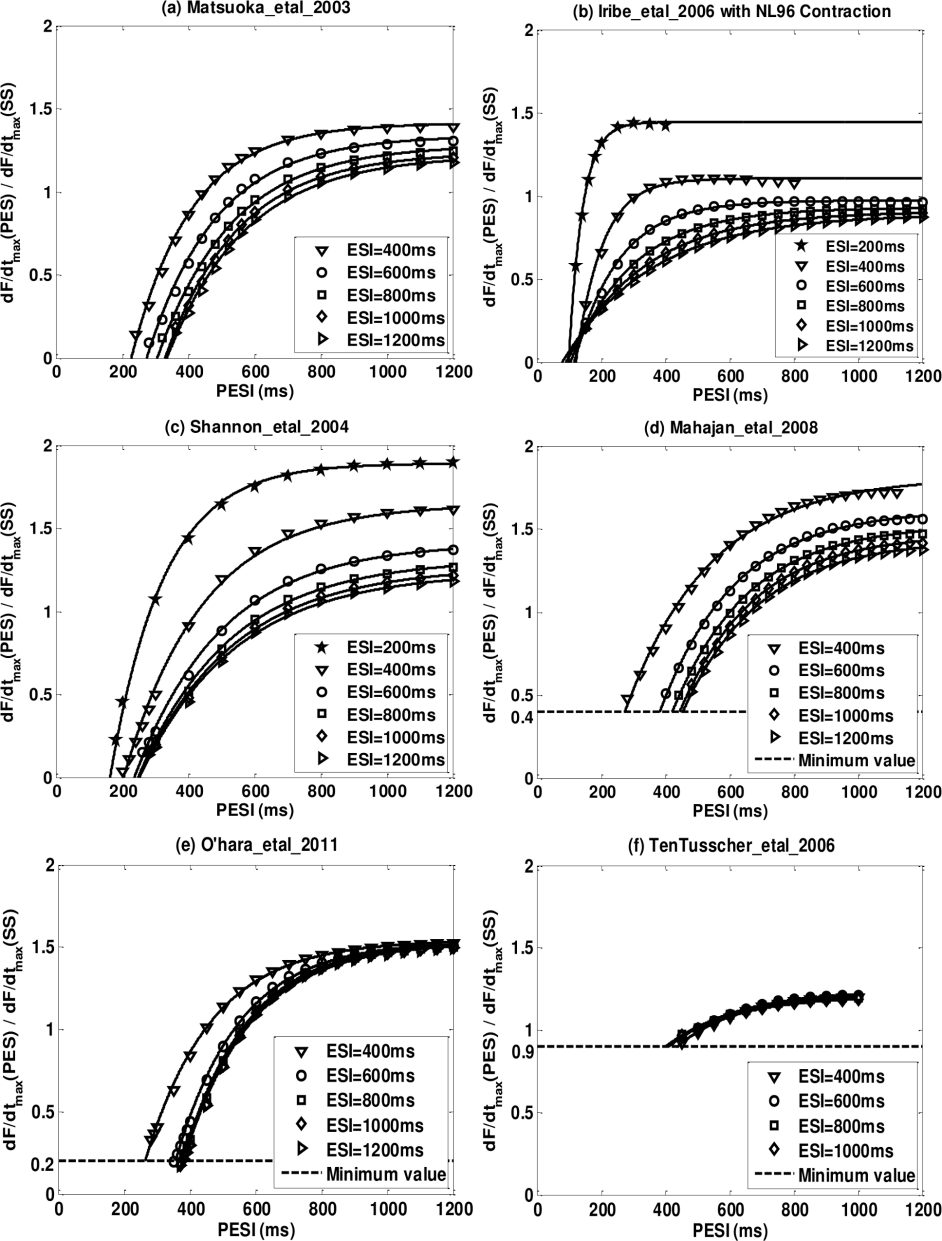
Representative postextrasystolic mechanical restitution curves (*MRC*_*pes*_) for six models after implementation of the NL96 contraction. (a) Matsuoka_etal_2003; (b) Iribe_etal_2006; (c) Shannon_etal_2004; (d) Mahajan_etal_2008; (e) O’hara_etal_2011; (f) TenTusscher_etal_2006.

Representative examples of *MRC*_*pes*_ (*dF* / *dt*_max_ (*PES*) / *dF* / *dt*_max_ (*SS*)) for six models are shown in Fig 6. For all the models, *MRC*_*pes*_ are given in S4 File.

**Fig 7:**
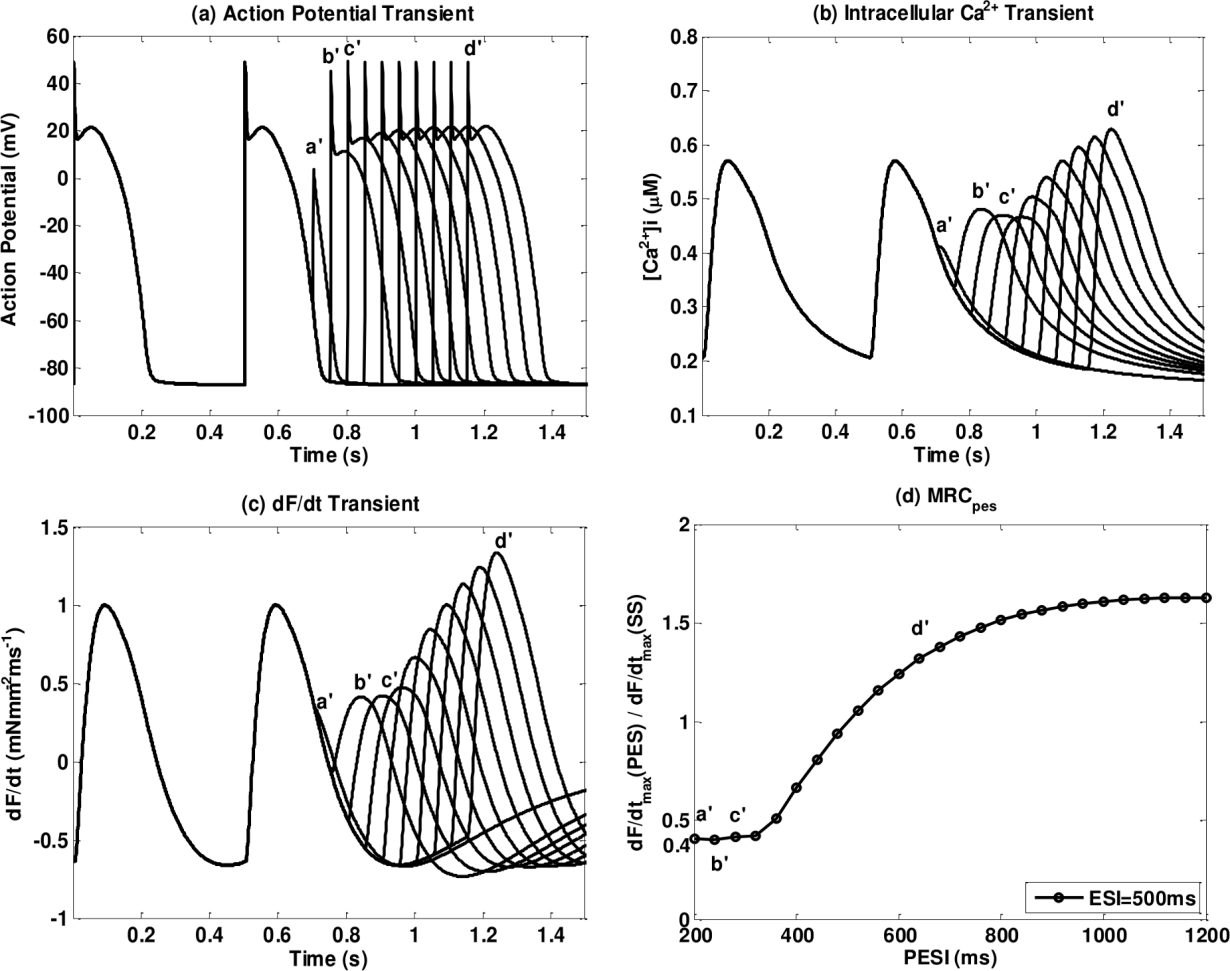
Details for cases where *C*_0_ does not go to zero. We use as example the Mahajan_etal_2008 for ESI = 500ms: (a) the action potential (AP), (b) [*Ca* ^2+^]_*i*_ transient; (c) normalized *dF* / *dt* transient (d) normalized *dF* / *dt*_max_ of the PES beats. In (a) (b) and (c), the first beat is the priming beat; the second beat is the ES beat with ESI = 500ms; from *a*' to d' are PES beats with PESI ranges from 200ms to 650ms.

For all models *MRC*_*pes*_ is well described (r^2^>0.99; averaged over all ESI values) by a monotonically increasing curve with respect to PESI (Eq 15) as shown in Table 8, after the elimination of some data points (see the example of Mahajan_eral_2008 below). However not all models were consistent with the experimental findings of Yue et al.[22] whose *C*_0_ = 0. Among the six models with dynamic *Ca* ^2+^ troponin buffers (Type One, Two, Three), four satisfy this condition, while among the eight models with instantaneous or none *Ca* ^2+^ troponin buffers (Type Four and Five), only one satisfies *C*_0_ = 0 (see Table 8). Here we provide a brief explanation why *C*_0_ does not go to zero for some models in our simulations. We use Mahajan_etal_2008 with NL96 as an example to demonstrate. In Fig 7(A), 7(B) and 7(C) we plot the Action Potential (AP), [*Ca* ^2+^]_*i*_ transient and normalized *dF* / *dt* transient of Mahajan_etal_2008 with NL96 for ESI = 500ms. The first beat is the steady priming beat, the second beat is the ES beat with ESI = 500ms and from *a*' to *d*' are PES beats with PESI from 200 to 650ms. We can observe that the *dF* / *dt*_max_ increases with PESI from beat *c*' to *d*' but there is no well-defined trend before *c*'. Beat *a*' and *b*' are not clearly separated from the ES beat and *a*' is even almost fused into the ES beat. However, even though beat *a*' is very close to the ES beat, *dF* / *dt*_max_ still does not go to zero. We plot normalized *dF* / *dt*_max_ (normalize to steady priming beat) for ESI = 500ms and PESI ranges from 200ms to 1200ms in Fig 7(D). We can see that *dF* / *dt*_max_ stops decreasing when PESI is below 300ms and the minimum value is about 0.4. In order to fit this into Eq 15 we exclude data with PESI lower than 300ms and set *C*_0_ = 0.4. That is the reason for Mahajan_etal_2008 and other eight models, that *C*_0_ does not go to zero and also an example of how we choose the shortest ESI and PESI values.

**Table 8:** Contraction parameters: MRC_pes_ and PESPC.

Model Type	Model Name	MRC_pes_ Mean r square	MRC_pes_ C_0_	PESPC_B	PESPC_A	PESPC_t_o,es_ (ms)	PESPC_T_pespc_ (ms)	PESPC r square
Experiment	Yue et al 1985 •	——-	0	1.05±0.13	1.68±0.32	284±32	176±18	——-
Type 1	Matsuoka_etal_2003	0.9967	0	1.13±0.05	0.34±0.04 XX	260±6	704±156 XX	0.9944
	Iribie_etal_2006	0.9953	0	0.90±0.01 **	1.01±0.09 **	119±6 XX	184±13	0.9969
Type 2	Shannon_etal_2004	0.9988	0	1.22±0.01 **	0.83±0.02 ***	219±6 **	263±9 XX	0.9996
	Grandi_etal_2010	0.9991	0	1.47±0.01 XX	0.65±0.02 XX	297±4	221±13 **	0.9983
Type 3	Mahajan_etal_2008	0.9987	0.4 XX	1.00±0.03	0.47±0.03 XX	344±6 **	346±49 XX	0.9938
	Iyer_etal_2004	0.9987	0.4 XX	1.49±0.05 XX	0.48±0.05 XX	366±3 ***	608±96 XX	0.9933
Type 4	Hund_etal_2004	0.9991	0	1.47±0.01 XX	0.02±0.01 XX	233±4 **	471±320 X	0.9043†
	Faber_etal_2000	0.9986	0.7 XX	0.74±0.01 ***	-0.01±0.01 XX	334±6 **	323±46 XX	0.9865†
	Livshitz_etal_2007	0.9998	0.5 XX	0.80±0.01 **	0.04±0.01 XX	312±1	413±100 XX	0.9814†
	Ohara_etal_2011	0.9988	0.2 XX	1.33±0.01 ***	0.02±0.01 XX	326±3 **	322±196 X	0.8848‡
	Priebe_etal_1998	0.9992	1.1 XX	0.77±0.01 ***	-0.00±0.01 XX	566±4 XX	232±7809 X	0.0048‡
Type 5	Fox_etal_2002	0.9991	0.5 XX	1.32±0.01 **	0.01±0.01 XX	321±4 **	328±94 XX	0.9156†
	TenTusscher_etal_2006	0.9971	0.9 XX	0.01±32.8 X	0.33±32.8 X	423±9 XX	1.04e4±1e6^X^	0.4278‡
	Fink_etal_2008	0.9977	0.9 XX	0.31±0.01 XX	0.01±0.01 XX	392±6 XX	190±121 X	0.8208‡

** Model’s 95% confidence bound overlaps the mean±2*SD value in Yue et al 1985 experiment

***Model’s 95% confidence bound overlaps the mean±3*SD value in Yue et al 1985 experiment

XX Model’s 95% confidence bound does not overlap the mean±n*SD value in Yue et al 1985 experiment with n≤3

X Model’s 95% confidence bound overlaps the mean±SD value in Yue et al 1985 but the bound is more than 50% of the center value

† 0.9 ≤r^2^ ≤0.99

‡ r^2^ <0.9

• Plus/minus is standard deviation. The other plus/minus are 95% confidence range

The experimental results of Yue et al.[22] showed that the fully restituted plateau value of *MRC*_*pes*_ (*CR*_max,*pes*_) decreased as ESI increased. From Fig 6 and S4 File we can see that all models with dynamic *Ca* ^2+^ troponin buffers show unique plateau values for different ESIs while models with instantaneous or no *Ca* ^2+^ troponin buffers converge to the same plateau values for different ESIs; this property will be further discussed in the PESPC subsection.

The time constant for *MRC*_*pes*_ (*T*_*mrc*,*pes*_) did not vary much as a function of ESI in the experiment of Yue et al.[22]. In addition, a leftward shift of the *MRC*_*pes*_ was observed as ESI decreased, presented as an increase of *t*_*o*,*pes*_ as ESI increased. The fourteen models behave differently regarding these two parameters. We will discuss these in detail in the *t*_*o*,*pes*_ and *T*_*mrc*,*pes*_ subsections.

#### Postextrasystolic Potentiation Curve (PESPC)

**Fig 8:**
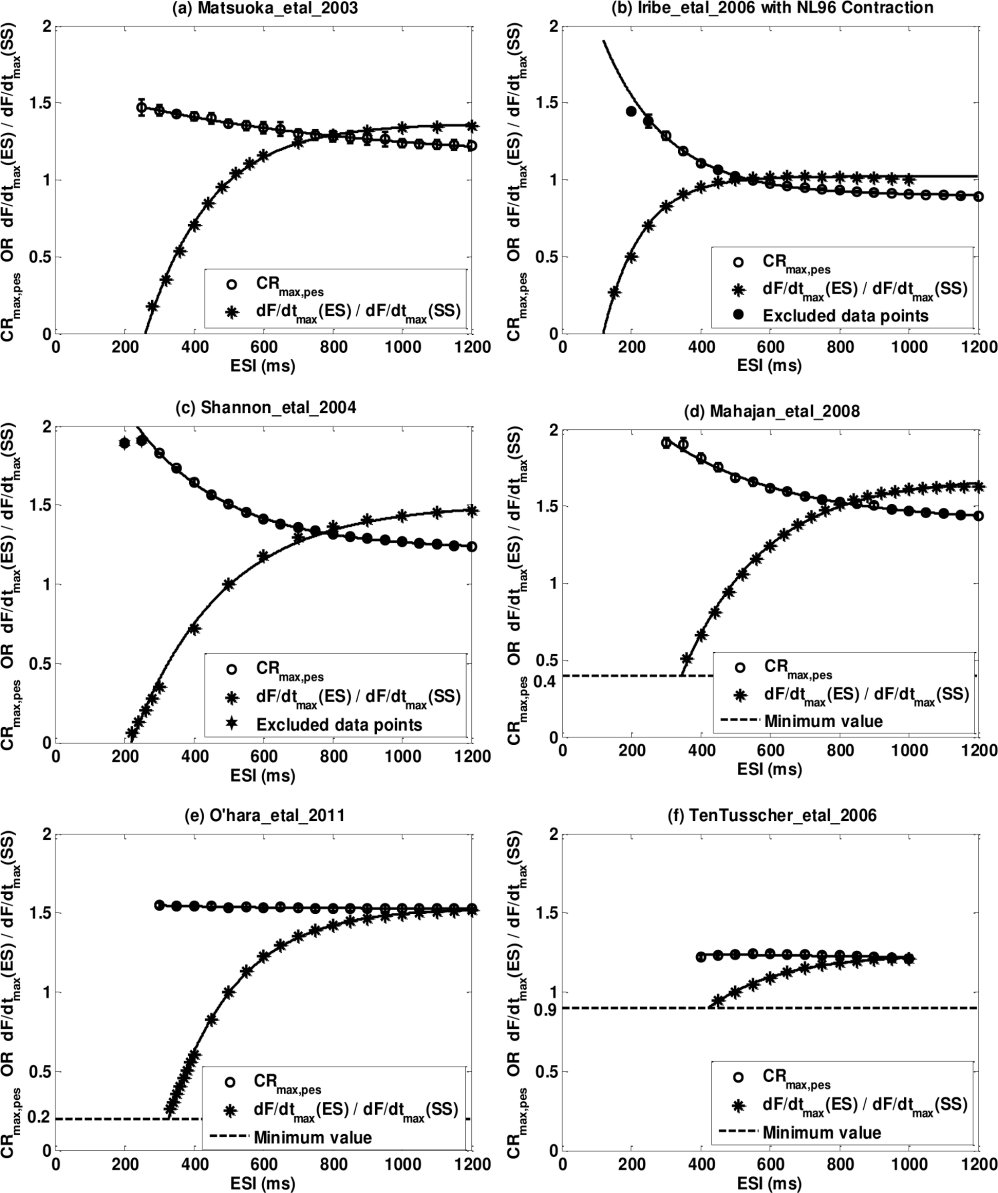
Representative postextrasystolic potnetiation curve (PESPC, circles o) and extrasystolic mechanical restitution curve (*MRC*_*es*_, stars *) for six models. (a) Matsuoka_etal_2003; (b) Iribe_etal_2006; (c) Shannon_etal_2004; (d) Mahajan_etal_2008; (e) O’hara_etal_2011; (f) TenTusscher_etal_2006. 95% confidence bounds of *CR*_max,*pes*_ are plotted as error bars on PESPC but since the confidence bounds are small compared to the entire scale, they are not clearly observed in the figures.

Fig 8 shows representative postextrasystolic potentiation curves (PESPCs) for six models. Figures for all models can be found in S5 File. To provide a better picture of the amplitude and time constant of the PESPC we simultaneously plot the PESPC and the Extrasystolic Mechanical Restitution Curve (*MRC*_*es*_) as in Yue et al.[22]. Note that for those models with *C*_0_ ≠ 0 in the *MRC*_*pes*_, we have shifted the curves upward by *C*_0_ when plotting the PESPC (star *) on the same graph with the *MRC*_*es*_ (open circle o) for comparison. We also use 95% confidence bounds as error bars for each data point on PESPC, which is the fitting confidence bound for *CR*_max,*pes*_ of each ESI value. Note that confidence bounds are relatively small compared to the scale of the graph.

A list of all parameters for the curve fitting of Eq 16 are listed in Table 8 for all the models along with the experimental data of Yue et al.[22] to allow comparison between our simulation results and those from the experiments. The values without any symbol match the experiment results well. They are either parameters with 95% confidence bounds overlapping the mean plus/minus standard deviation (mean±SD) range from Yue et al.[22] or they have r^2^ values that are higher than 0.99. The double and the triple star (** and ***) means the 95% confidence bound overlaps Yue et al.[22] data’s mean±2*SD and the mean±3*SD respectively. The X mark means the confidence bound overlaps the mean±SD but the confidence bound is too large (more than 50% of the center value) so that we do not consider this as a real overlapping. The double X mark (XX) means the confidence bound does not overlap the mean±n*SD from Yue experiment with n≤3.The single-dagger (†) means the r^2^ is less than 99% but more than 90% and the double-dagger (‡) means it’s less than 90%.

The experiments of Yue et al.showed that PESPC decreased monoexponentially to a plateau level as ESI increases (Eq 16 and Fig 1(B)), meaning that when PESI is fixed and long enough, the maximum force changing rates of the PES beats will decrease as ESI increases. Models with dynamic *Ca* ^2+^ troponin buffers (Type One, Two and Three) have PESPCs well described by Eq 16; all six models have r^2^ values larger than 0.99. On the other hand, models with instantaneous or no *Ca* ^2+^ troponin buffers (Type Four and Five) are not well fit by Eq 16. Four of the eight models have r^2^ values lower than 0.9 with one of them as low as 0.0048 (Priebe_etal_1998). However, the poor quality of the fitting is not evident on Fig 8 or S5 File because the amplitude of the PESPC is much smaller than the scale of the figure.

**The plateau levels (*B*) of *CR***_**max,*pes***_ for the fourteen contraction models are more similar to the experiment results compared to the other parameters. In Yue et al.[22], the mean value (± SD) for *B* is 1.05±0.13. In our simulations, two out of the six models with dynamic *Ca* ^2+^ troponin buffers (Type One, Two and Three) have 95% confidence bounds overlap the mean±SD range of Yue et al.[22] and four have confidence bounds overlap the mean±2*SD range. On the other hand, only two out of eight models with instantaneous or no *Ca* ^2+^ troponin buffers (Type Four and Five) have 95% confidence bounds overlap the mean±2*SD range.

**Parameter *A*** has the least matching degree with Yue et al.[22] where the mean value (± SD) is 1.68±0.32. Only one out of the six models with dynamic *Ca* ^2+^ troponin buffers (Type One, Two and Three) have 95% confidence bounds overlap the mean±2*SD range from Yue paper and none of the models with instantaneous or none *Ca* ^2+^ troponin buffers have confidence bounds overlapping this range (see Table 8). The values of *A* from our contraction models are significantly smaller than Yue paper. This feature is well depicted in Fig 8 and S5 File in which the PESPCs of the models with instantaneous buffers have slopes of approximate zero while in the Yue et al. paper there exhibited a pronounced monoexponentially decay (Fig 1(B)). The insufficient change in fully-restituted contractile strength of postextrosystoles with respect to varied ESIs indicates a clear limitation of these models as we will further comment in the discussions section.

**t**_**o,es**_ is obtained by calculating where the Extrasystolic Mechanical Restitution Curve (*MRC*_*es*_) intercepts the line $\frac{dF\;/\;dt_{\text{max}}\;(ES)}{dF\;/\;dt_{\text{max}}\;(SS)}=C_{0}$. The mean value (±SD) in the Yue et al.[22] is 284±32ms. Four out of six models with dynamic *Ca* ^2+^ troponin buffers (Type One, Two and Three) and five out of eight models with instantaneous buffers (Type Four and Five) are in the mean±2*SD range. For this parameter, it *seems* that the dynamic buffers do not show much advantage over instantaneous buffers. However, in the Yue et al. paper, this interception is where *MRC*_*es*_ intercepts with the line $\frac{dP\;/\;dt_{\text{max}}\;(ES)}{dP\;/\;dt_{\text{max}}\;(SS)}=0$, so are four out of the six models with dynamic *Ca* ^2+^ troponin buffers. But for most models with instantaneous buffers, this parameter is where *MRC*_*es*_ intercepts with the line $\frac{dF\;/\;dt_{\text{max}}\;(ES)}{dF\;/\;dt_{\text{max}}\;(SS)}=C_{0}$ with *C*_0_ ≠ 0. Therefore models with dynamic buffers still match this parameter better to the experimental data of Yue et al.[22].

The mean value (± SD) of the **time constant for PESPC (*T***_***pespc***_ **)** is 176±18ms in Yue et al.[22]. Only two out of the six models with dynamic *Ca* ^2+^ troponin buffers (Type One, Two, Three) and none of the models with instantaneous or none buffers (Type Four and Five) have 95% confidence bounds that overlap the experimental mean±2*SD range. Ten other models have time constants much larger(50% more) than Yue et al.[22]. This parameter is also the least well fit for models with instantaneous or no *Ca* ^2+^ troponin buffers; five out of eight models have 95% confidence bounds larger than 50% of the center values, indicating a poor fit.

#### Minimum-value Axis Intercept Curve (*t*_*o*,*pes*_)

**Fig 9:**
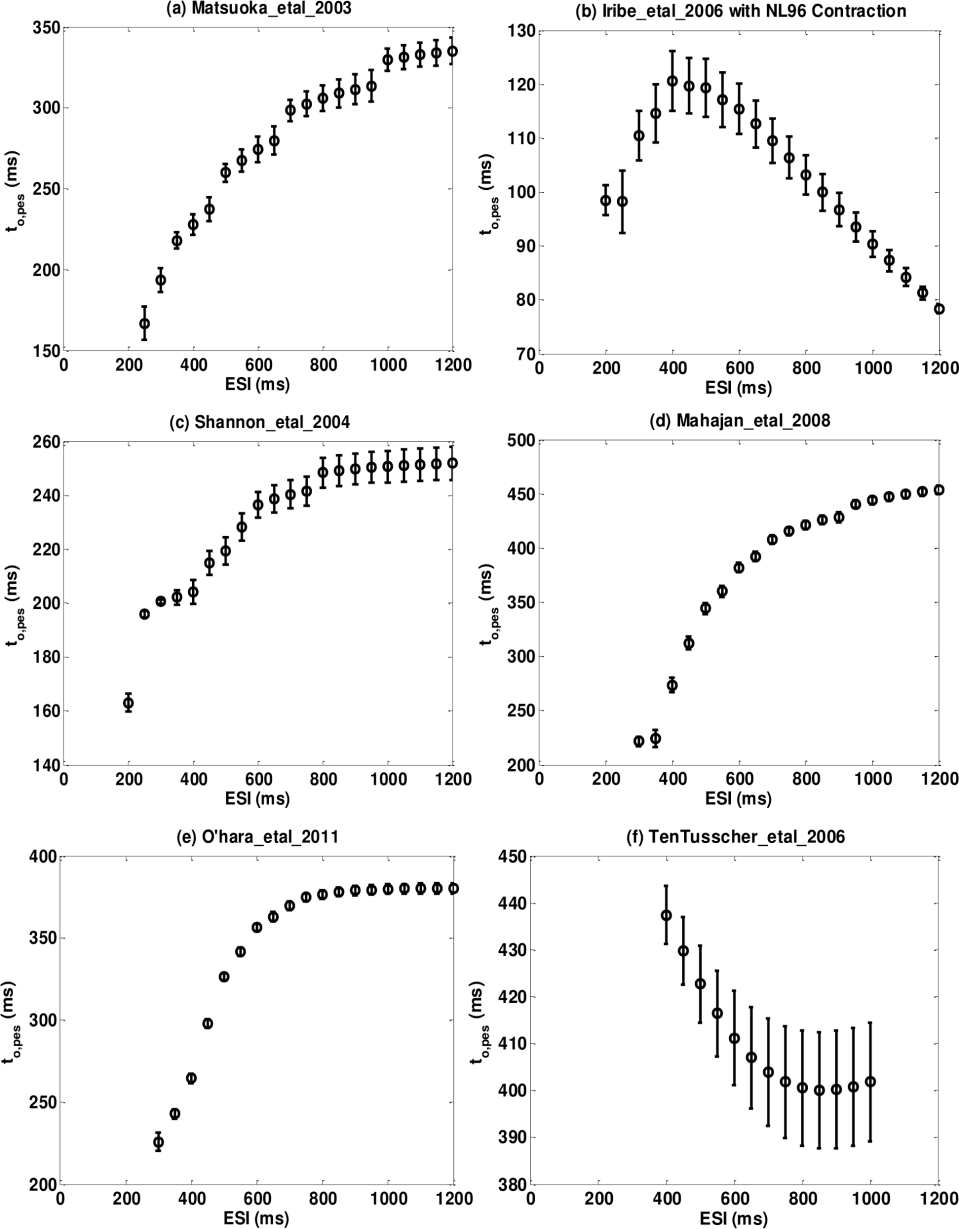
Representative minimum-value axis intercept curve (*t*_*o*,*pes*_) for six models. (a) Matsuoka_etal_2003; (b) Iribe_etal_2006; (c) Shannon_etal_2004; (d) Mahajan_etal_2008; (e) O’hara_etal_2011; (f) TenTusscher_etal_2006. 95% confidence bounds of *t*_*o*,*pes*_ are plotted as error bars.

Fig 9 shows Minimum-value Axis Intercept Curves for six models. Open circles (o) are *t*_*o*,*pes*_ for different ESI values. Error bars are 95% confidence bounds. Figures for all models are provided in the S6 File and the summarized data is in Table 9.

**Table 9:** Contraction parameters: t_o,pes_ and T_mrc,pes_.

Model name	t_o,pes_		T_mrc,pes_		
	Trend w.r.t. ESI	Range(ms)	T_mrc,es_(ms)	Mean±SD •	T_pespc_-T_mrc,es_(ms)
Yue_etal_1985 •	Monotonic increase	180–420	181±41	1.01±0.12	13±23
Matsuoka_etal_2003	Monotonic increase	167–335	187±13	1.15±0.12	517±156 XX
Iribie_etal_2006	Not monotonic XX	78–120 XX	110±9 **	1.52±0.71	74±13 ***
Shannon_etal_2004	Monotonic increase	163–252	262±16 **	0.98±0.17	1±16
Grandi_etal_2010	Monotonic increase	245–350	234±10 **	1.01±0.11	-13±13
Mahajan_etal_2008	Monotonic increase	221–454	238±14 **	1.04±0.08	108±49 **
Iyer_etal_2004	Monotonic increase	305–498	333±10 XX	1.04±0.04	275±96 XX
Hund_etal_2004	Monotonic increase	126–242	234±9 **	1.00±0.02	237±320 ※
Faber_etal_2000	Monotonic decrease XX	297–342	300±13 ***	1.02±0.04	23±46
Livshitz_etal_2007	Monotonic increase	232–333	203±3	1.02±0.06	210±100 XX
Ohara_etal_2011	Monotonic increase	226–380	193±8	1.04±0.02	129±196 ※
Priebe_etal_1998	Not monotonic XX	377–624	438±14 XX	1.04±0.03	-206±7809 ※
Fox_etal_2002	Not monotonic XX	244–380	370±8 XX	1.00±0.02	-42±94 ※
TenTusscher_etal_2006	Monotonic decrease XX	402–437	209±25	1.04±0.04	1.02e4±1e6※
Fink_etal_2008	Monotonic decrease XX	341–420	256±18 **	1.06±0.03	-66±121 ※

** Model’s 95% confidence bound overlaps the mean±2*SD value in Yue et al 1985 experiment

*** Model’s 95% confidence bound overlaps the mean±3*SD value in Yue et al 1985 experiment

XX Model’s 95% confidence bound does not overlap the mean±n*SD value in Yue et al 1985 experiment with n≤3

※ Model’s 95% confidence bound overlaps the mean±SD value in Yue et al 1985 but the bound is more than 3*SD

• Plus/minus is standard deviation. The other plus/minus are 95% confidence range.

In the Yue et al. paper[22], *t*_*o*,*pes*_ increased as ESI increased. They explained this trend by suggesting that the refractory period is an increasing function of ESI[22]. Five out of six models with dynamic buffers (Type One, Two and Three) show the same trend as Yue et al. paper with *t*_*o*,*pes*_ increasing exponentially with ESI; only the Iribe_etal_2006 model shows a non-monotonic behavior. Three out of eight models with instantaneous buffers (Type Four and Five) show the same trend as Yue et al. (Hund_etal_2004, Livshitz_etal_2007 and O’hara_etal_2011) while three models show a monotonically *decreasing* trend (Faber_etal_2000, TenTusscher_etal_2003 and Fink_etal_2008) and two (Priebe_etal_1998 and Fox_etal_2002) do not have monotonic behaviors. The Ranges of *t*_*o*,*pes*_ of the models match Yue et al. paper well as thirteen out of fourteen models fall within the experimental range.

#### Time Constant Curve (T_mrc,pes_)

**Fig 10:**
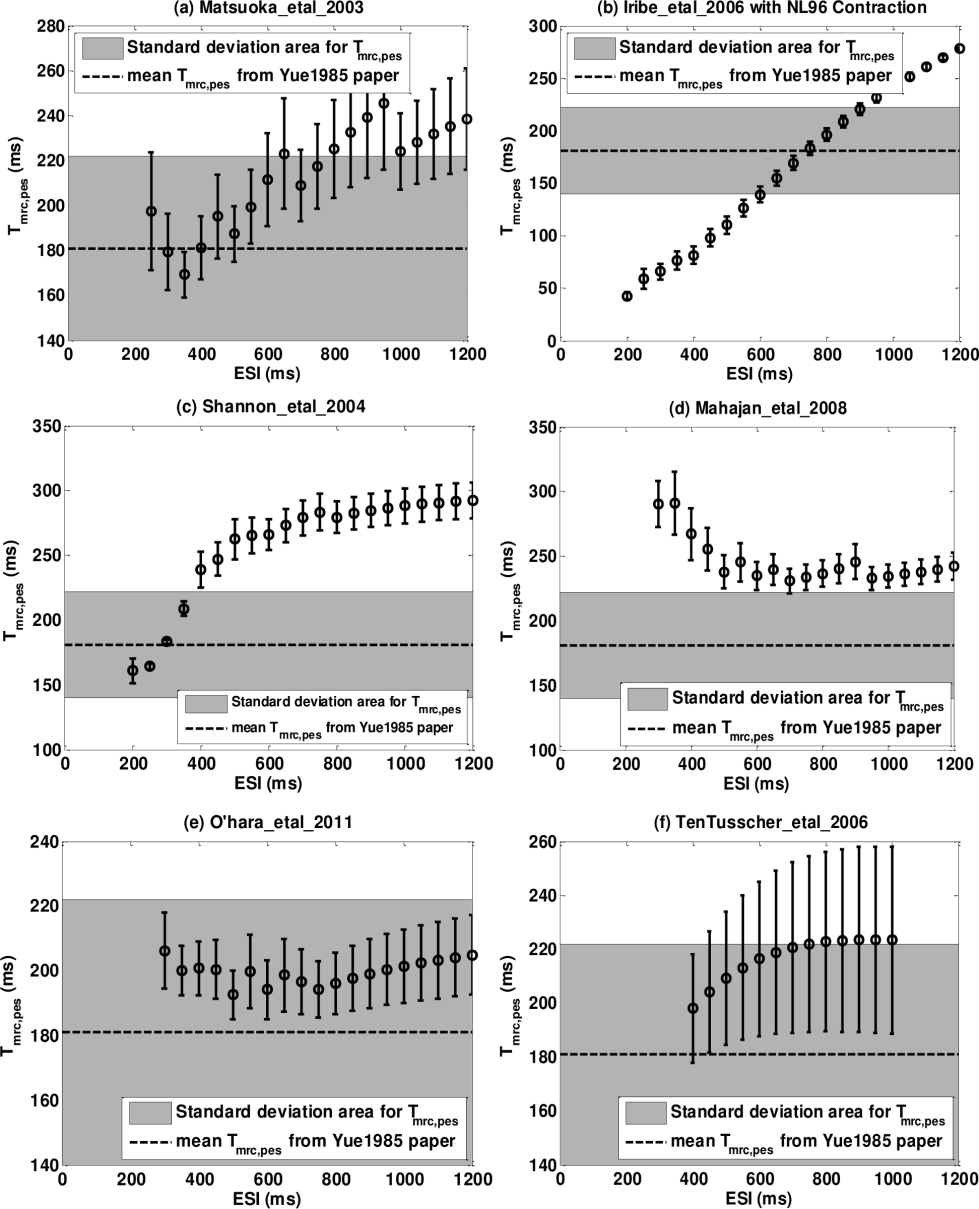
Representative time constant for *MRC*_*pes*_ curve (*T*_mrc,*pes*_) for six models. (a) Matsuoka_etal_2003; (b) Iribe_etal_2006; (c) Shannon_etal_2004; (d) Mahajan_etal_2008; (e) O’hara_etal_2011; (f) TenTusscher_etal_2006. Error bars are 95% confidence bounds. The dash line indicates mean *T*_*mrc*,*pes*_ for ESI = 460ms from Yue et al. The grey area denotes their standard deviation range.

Fig 10 shows six Time Constant Curves, i.e. time constants of *MRC*_*pes*_ (*T*_*mrc*,*pes*_) vs ESI for 6 models, where the dash line indicates the mean *T*_*mrc*,*es*_ from the Yue et al. paper; the grey area denotes the range of the mean±SD range. Open circles (o) are *T*_*mrc*,*pes*_ from curve fitting for different ESI values. Error bars are 95% confidence bounds. Figures for all the models are in S7 File. There were two major results in Yue et al.[22] regarding *T*_*mrc*,*pes*_. First, *T*_*mrc*,*pes*_ varied little with ESI; the mean (± SD) *T*_*mrc*,*es*_ was 181±41ms and the mean normalized *T*_*mrc*,*pes*_ (normalize to *T*_*mrc*,*es*_) was 1.01±0.12. In our simulations, all of the models have their normalized time constant values overlap the mean±2*SD range from Yue paper while ten of the models have their *T*_*mrc*,*es*_ (identical to *T*_*mrc*,*pes*_ with ESI = 500ms in simulations) overlap the mean±2*SD range from Yue paper. Therefore our contraction models fit this property with Yue paper quite well. The second major result from Yue paper is that the time constants of PESPC and *MRC*_*pes*_ were close; the mean difference between the two time constants (± SD) was 13±23ms. Models with dynamic *Ca* ^2+^ troponin buffers (Type One, Two and Three) are more consistent with the experiments than instantaneous buffers (Type Four and Five) in this respect. Three out of six models with dynamic buffers have 95% confidence bounds that overlap Yue’s mean±2*SD range while only one out of eight models with instantaneous buffers overlaps that. Six of them have large 95% confidence bounds inherited from the large *T*_*pespc*_ bounds, so we marked them by a symbol **※**, indicating the 95% confidence bounds overlap Yue’s mean±SD but the confidence bound is so large (more than 3*SD) that we do not consider this as a real overlapping.

### Underlying Mechanism for PESP

There is a clear resemblance between the intracellular *Ca* ^2+^ dynamic and force dynamics. And since the *Ca* ^2+^ released from SR (*J*_*rel*_) is the main source for intracellular *Ca* ^2+^, we propose that *J*_*rel*_ dynamics, which depends on RyR, $Ca_{SR}^{2+}$, among others, is the primary determinant of PESP dynamics.

**Fig 11:**
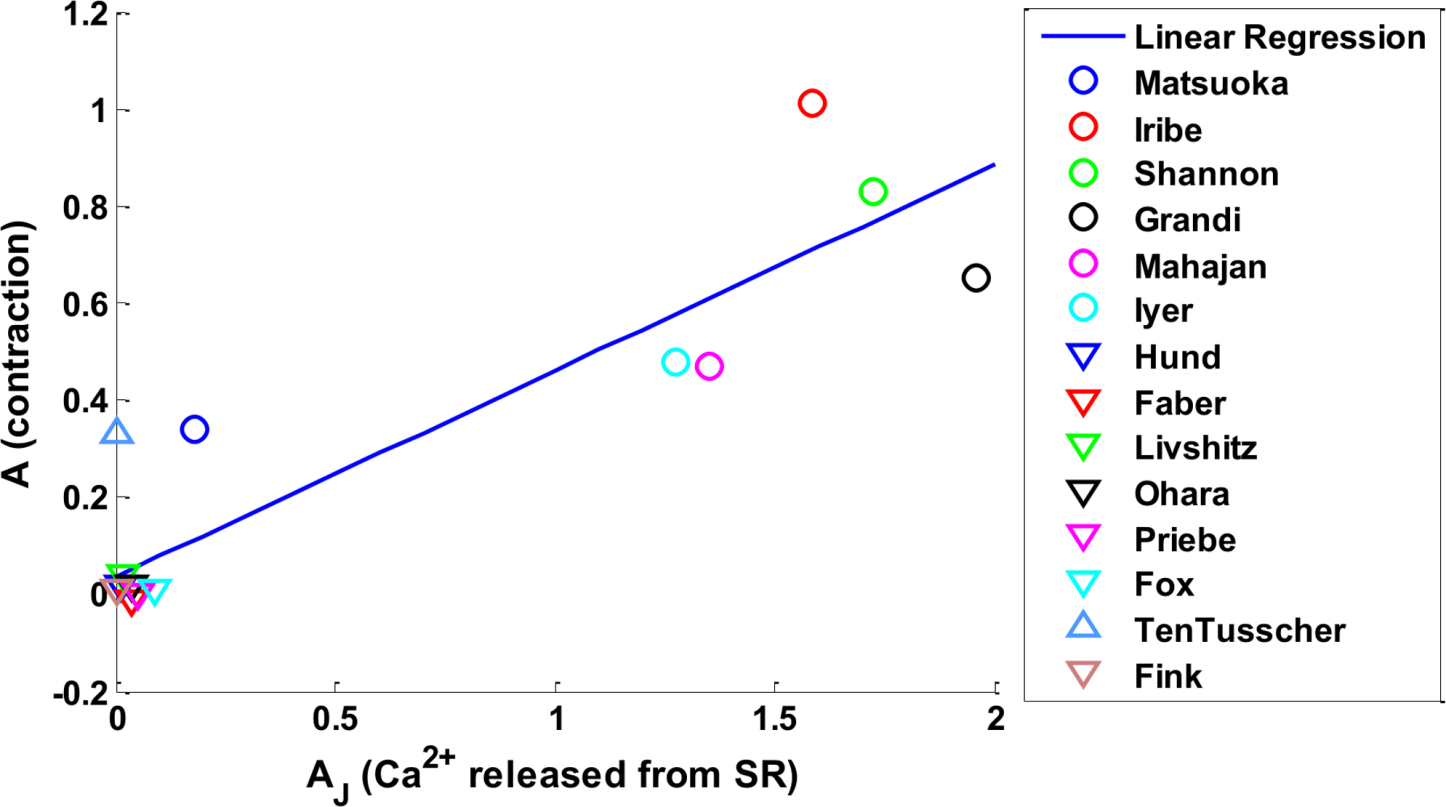
Correlation between the potentiation in Jrel and contractile strength. The Postextrasystolic Potentiation Curves (PESPC) amplitude of contractile strength (*A*) vs that of calcium released from SR (*A*_*J*_).

To verify this hypothesis, we analyzed, for each model, the correlation between the dependence of the potentiation in *Ca* ^2+^ released from SR (*J*_*rel*_) and the contractile strength on pacing intervals. In this study we have shown two sets of curves demonstrating the potentiation strength: the Postextrasystolic Mechanical Restitution Curve (*MRC*_*pes*_) and Postextrasystolic Potentiation Curve (PESPC). The *MRC*_*pes*_ demonstrates how postextrasystolic contractile strength changes with respect to various PESI for a fixed ESI. From Fig 6 and S4 File, it can be seen the consistency of the increasing trend of the normalized force changing rate with respect to PESI for all models studied. However, the PESPC, which shows how postextrasystolic contractile strength changes with respect to various ESI values for a fixed and long PESI, differs among models: models with dynamic buffers showed significant variation in contractile strength with respect to ESI while models with instantaneous buffers showed little variation (Fig 8 and S5 File). The amplitude (parameter *A*) in PESPC captures this difference. Since we are interested in what causes the difference in models, we studied the correlation between the *Ca* ^2+^ released from SR (*J*_*rel*_) and the contractile strength of the postextrasystoles with various ESI and fixed PESI. We designed an analogous parameter *A*_*J*_ for the *J*_*rel*_ amplitude. Similar to our *dF* / *dt*_max_ fit, for a fixed ESI, we first fit the normalized *J*_*rel*_max_ (to the prime beats) of the postextrasystolic beats to a mono-exponentially increasing curve with respect to PESI (analogous to *MRC*_*pes*_). For most models, *J*_*rel*_max_ was well described by a mono-exponential increasing curve, similar to the contractile strength. Then we fit the plateau values of these curves to a mono-exponentially decreasing curve with respect to ESI (analogous to PESPC); we define the amplitude of this curve as *A*_*J*_. Models with larger *A*_*J*_ suggest that their fully-restituted *J*_*rel*_max_ vary with respect to ESI. Therefore the correlation between *A* and *A*_*J*_ can be used as a gauge for the correlation between the dependence of *J*_*rel*_ and the contractile strength on pacing intervals. We performed a linear regression for *A*_*J*_ and *A* for thirteen (out of the fourteen) electromechanical models as shown in Fig 11. We found a strong positive correlation (r^2^ = 0.848) between the two parameters. In addition, data from models with instantaneous buffers clustered around the origin while data from models with dynamic buffers were distributed along the regression curve. **This means for models with instantaneous buffers, the insufficient variation in the fully-restituted postextrasystolic contractile strength responding to various ESI is a direct result of the *J***_***rel***_ **dynamics.** Note that the model excluded from the linear regression was TenTusscher_etal_2006 (upward triangular mark in Fig 11) because the curve fitting for parameter *A* in this model is not reliable due to its large 95% confidence bound (see Table 8).

## Discussion

Simulations of electrical therapies for HF (e.g., CRT and CCM) require models that accurately reproduce the excitation-contraction coupling that occurs in the cardiac myocyte. While action potential duration restitution curves tend to be used to help validate EP models, there has been no systematic method to validate cellular contraction models. We suggest that the PESP protocol can be used in the validation process of mathematical models of cellular electromechanics and we present a methodology to include mechanics into cellular EP models of various types and compare results among the models with and without contraction.

The results from this study suggest four reasons for which electrophysiology models with dynamic *Ca* ^2+^ troponin buffers are much better candidates to implement contraction than EP models with instantaneous or none *Ca* ^2+^ troponin buffers.

First, the complexity in implementation is greatly reduced since the original models are already equipped with dynamic equations for *Ca* ^2+^ troponin buffers. Type Two and Type three models only take one step in the implementation flowchart while Type Four and Type Five require one or two more steps to add in instantaneous *Ca* ^2+^ troponin buffers and change the instantaneous ones into a dynamic form (see S1 File).

Second, the inclusion of contraction does not disturb properties in the original models. The [*Ca* ^2+^]_*i*_ shape shows minimum change for models with dynamic buffers while it is largely distorted for models with instantaneous or none *Ca* ^2+^ troponin buffers (Fig 2 and S2 File). This is closely related to the first benefit as it requires adding extra terms into Type Four and Type Five models to incorporate contraction and therefore *Ca* ^2+^ shapes are more likely to be unphysiologically deformed.

Third, models with dynamic *Ca* ^2+^ troponin buffers show clear postextrasystolic potentiation in [*Ca* ^2+^]_*i*_ with or without contraction implemented (see Figs 4 and 5 and S3 File). Therefore these models show desired variation in contractile strength of extrasystoles and postextrasystoles because of the close relationship between *Ca* ^2+^ and contraction.

Fourth, contraction properties of models with dynamic *Ca* ^2+^ troponin buffers fit experimental results better [22] (see Figs 6, 8, 9 and 10). This has been shown in the four characteristic curves (Postextrasystolic Mechanical Restitution Curve (*MRC*_*pes*_); Postextrasystolic Potentiation Curve (PESPC); Minimum-value Axis Intercept Curve (*t*_*o*,*pes*_) and Time Constant Curve (*T*_*mrc*,*pes*_)) and their various parameters.

To unveil the mechanism why model with dynamic buffers reproduce PESP better than models with instantaneous buffers, we studied the correlation between contraction and calcium release from SR. We found a strong positive correlation (r^2^ = 0.848). This means for models with instantaneous buffers, the insufficient variation in contractile strength responding to various pacing cycle length is a direct result of the *J*_*rel*_ dynamics. The interpretation for this result is twofold. First, *Ca* ^2+^ release from SR is the main source for intracellular *Ca* ^2+^ therefore *J*_*rel*_ can be the direct reason of contraction behaviors. Second, time constants play a crucial role in cell contraction. The time constants of *Ca* ^2+^ binding with troponin C in Eq 6 in models with dynamic buffers are in the order of 10ms, which is the same order of the time scales of the upstroke of *Ca* ^2+^, *CaTRPN* and tension development. The RyR release kinetics is highly timing sensitive and strongly coupled to the *CaTRPN* binding process. Therefore models with dynamic buffers, which can describe the timing of interaction among different cell compartments more accurately, reproduce correct contraction behaviors. This has biological significance since most electrophysiological models incorporate instantaneous buffering due to their simplicity and lower computational cost.

To assess the robustness of the contraction model we use, we conducted more careful verifications in the supplementary material S8 and S9 Files.

In S8 File, we compared NL96 with two other contraction models: i) NL96 and RWH99 implemented in Iribe_etal_2006; ii) dynamic (original) NL96 and instantaneous NL96 implemented in Ohara_etal_2011. In the first set of comparison, we conclude that under the isometric condition there are no significant differences between NL96 and RWH99 under the scope of this study in the sense that each one matches different parameters with experiments better than the other. In the second set of comparison we find that although including NL96 in models with instantaneous *Ca* ^2+^ troponin buffers distort their *Ca* ^2+^ shape, the poor contraction behaviors of these models are not directly related to that. Two simulation results support this conclusion. First, their original models do not show clear postextrasystolic potentiation behavior in [*Ca* ^2+^]_*i*_ (see Fig 5), which is directly related to the lack of variation in contractile strength and second, *Ca* ^2+^ shape is retained after the implementation of the instantaneous NL96 yet the contraction behavior of Ohara_etal_2011 is still not optimal.

Finally in S9 File, we conducted verifications of physiological parameters such as different priming period, low temperature and cooperativity of the contraction model. By comparing simulation results before and after modification of these physiological parameters, we showed that the choice of parameters did not change quantitatively the PESP behavior.

### Limitations and future work

Yue’s experiments are based on dog ventricles in iso-volume conditions, but our simulations are on isometric single cells. However similar experiment results have been observed in smaller tissue sizes. Wier et al. [28] measured tension in perfused ferret papillary muscles 0.67±0.05mm in external diameter and reported similar monoexponential curves as Yue et al.. We do consider the size to be a potential limitation of our work. Qualitatively we get similar characteristic curves in our simulations as in the experiment but quantitatively none of our models show as much variation in contraction strength with respect to changing stimulus rate (see Figs 6 and 8) as in Yue’s experiment. One possible reason might be that the coupling and isotropy in tissues reinforce the variation in contractile strength, which suggests tissue simulations should be one direction for future work.

Another limitation on using some of the cell models is the coupling of cross species models and the comparison to experimental data from another species. The argument is that, first, the parameters in the experiment and simulations that we performed quantitatively cross species comparisons on are all normalized to their own priming beats (Figs 6 and 8); the normalization should reduce the species sensitive factors. Second, the quantities that are not normalized are either compared before and after the contraction implementation within each own individual model (Figs 2, 4 and 5) or compared qualitatively with the experiment (trends with respect to ESI in Figs 9 and 10). Nevertheless we admit this is a limitation in our work due to the lack of experiment data for all species.

In our study, we chose a hybrid approach to implement contraction where we only changed CaTRPN into a dynamic buffer while kept all the other buffers instantaneous. The reason for that is because we wanted to modify the original model as little as possible. Since only CaTRPN is presented in the contraction model, we changed only that into a dynamic buffer. We however did conduct simulations on O’Hara et al model to investigate the effect of dynamic changes in other buffers, specifically we changed both of the instantaneous intracellular calcium buffers in the model (troponin and calmodulin) into dynamic buffers using the same strategy described in the Method section. We found that doing these changes not only affected the calcium as before but in addition the voltage AP, both by large amount (not shown).

## Supporting Information

S1 File Flowchart of the implementation of NL96 corresponding to different types of models. (DOC)

S2 File Priming [*Ca* ^2+^]_*i*_ transients for all models. (DOCX)

S3 File Postextrasystolic potentiation in [*Ca* ^2+^]_*i*_ for all models. (DOCX)

S4 File Postextrasystolic mechanical restitution curves (*MRC*_*pes*_) for all models. (DOCX)

S5 File Postextrasystolic potnetiation curve (PESPC, circles o) and extrasystolic mechanical restitution curve (*MRC*_*es*_, stars *) for all models. (DOCX)

S6 File Minimum-value axis intercept curve (*t*_*o*,*pes*_) for all models. (DOCX)

S7 File Time constant of *MRC*_*pes*_ curve (*T*_mrc,*pes*_) for all models. (DOCX)

S8 File Comparison between NL96 and two other contraction models. (DOCX)

S9 File Varying physiological parameters. (DOCX)
