# Supplementary material for: Implementation of Contraction to Electrophysiological Ventricular Myocyte Models, and Their Quantitative Characterization via Post-Extrasystolic Potentiation
Source: PLoS One. 2015 Aug 28;10(8):e0135699. doi: 10.1371/journal.pone.0135699 (PMC4552858; doi:10.1371/journal.pone.0135699)
Supplement: S1 File — (DOC) [file pone.0135699.s001.doc]

**Model has contraction?**

**Model has full dynamic buffers?**

**Model has dynamic CaTRPN?**

**No**

**Model has CaTRPN buffer?**

**No. Type 5**

***Step 1****:* **Add instantaneous CaTRPN buffer**

**Yes. Type 1**

**Yes. Type 2**

**Yes. Type 3**

**Yes. Type 4**

**Step 0: Take out contraction**

***Step 3:* Split the single state dynamic CaTRPN in Step 2 (or original model) into 2 states: with (TCa*) or without (TCa) cross-bridges.** **Keep all the other buffers unchanged.**

**No**

**No**

***Step 2:*** **Change the instantaneous intracellular CaTRPN into a dynamic buffer.**

***Step 0:* Handling models that already have contractions.**

If the model has NL96 contraction then we only need to take that out.

For example, Matsuoka_etal_2003 has NL96 contraction. We eliminate the four troponin states in NL96 and replace them with the single-state dynamic equation for CaTRPN like Eq(9) and set and to the values of and $Z1$.

If the model has contraction but not the NL96 one, we first take out the original contraction then implement NL96. For example, Iribe_etal_2006 model has Rice1999 contraction. Rice mechanics model has six tropomyosin/cross-bridge states. The transform rates among these six states are related to CaTRPN. Their CaTRPN is expressed by a single-state dynamic equation like Eq(9) with a dynamic $K_{off}$ dependent on force. To take out contraction from Iribe_etal_2006, we eliminate the six tropomyosin/cross-bridge states and set $K_{off}$ for CaTRPN to a constant value by fixing the force in the $K_{off}$ expression to half of its maximum value. After taking out Rice1999 contraction, follow Step 3 in the ‘NL96 contraction implementation’ flowchart to implement NL96.

***Step 1****:* **Add instantaneous CaTRPN buffer**

If the model has one general Ca^2+^ buffer (referred as General) representing the average effect of all intracellular Ca^2+^ buffers (e.g. TenTusscher_etal_2006 and Fink_etal_2008), we split the general buffer into two parts: CaTRPN and Others. We keep and to be the same as in the original model so the affinity of the instantaneous buffer will be retained. is set to be 0.07mM, which is a standard value in multiple models. so that the concentration of the total intracellular Ca^2+^ buffer doesn’t change.

If the model has other specified Ca^2+^ buffers (such as CMDN) but no CaTRPN (e.g. Fox_etal_2002), we keep the other specified buffers unchanged and add a CaTRPN buffer. For the new CaTRPN buffer we set $[Troponin]_{total}=0.07mM$and $K_{dCaTRPN}=0.6\mu M$which are both standard values.

***Step 2:*** **Change the instantaneous intracellular CaTRPN into a dynamic buffer**.

Keep all other buffers instantaneous.

1. Equations
2. Original:

$$\frac{d[Ca^{2+}]_{i}}{dt}=\beta I_{total}^{Ca}$$

$\beta=1/(1+\sum_{j} \frac{B_{\max,j}K_{d,j}}{([Ca^{2+}]_{i}+K_{d,j})^{2}})$ $j$: all intracellular Ca^2+^ buffers

1. Modified

$$\frac{d[Ca^{2+}]_{i}}{dt}=\beta'(I_{total}^{Ca}-I_{trop})$$

$\beta'=1/(1+\sum_{j} \frac{B_{\max,j}K_{d,j}}{([Ca^{2+}]_{i}+K_{d,j})^{2}})$ $j$: all intracellular Ca^2+^ buffers except for CaTRPN

$$I_{trop}=\frac{d[CaTRPN]}{dt}=K_{on}[Ca^{2+}]_{i}(B_{\max,troponin}-[CaTRPN])-K_{off}[CaTRPN]$$

In the above equations, $[Ca^{2+}]_{i}$ is intracellular Ca^2+^ concentration; $I_{total}^{Ca}$ is all the intracellular Ca^2+^ currents; *j* represents each type of intracellular Ca^2+^ buffers; and are total concentration and the affinity constant for buffer *j*. To change instantaneous CaTRPN into a dynamic buffer, we delete the term corresponding to troponin in the instantaneous buffer factor β and substract the CaTRPN current from the total intracellular Ca^2+^ currents. The dynamic equation for CaTRPN represents the simple chemical reaction:

The way to choose $K_{on}$ and is not unique as long as . We choose and $K_{on}=\frac{1}{\sqrt{\sigma}}K_{on,s}$. *s* indicates the values from Shannon_etal_2004 model and .

1. Initial conditions
2. and other variable values are quiescent values of the original model
3. is the value that makes , namely $[CaTRPN](t=0)=\frac{B_{\max,troponin}[Ca^{2+}]_{i}}{[Ca^{2+}]_{i}+K_{d,troponin}}$

***Step 3:* Split the single state dynamic CaTRPN in Step 2 (or original model) into 2 states: with (TCa*) or without (TCa) cross-bridges.** Keep all the other buffers unchanged.

1. Equations

$$\frac{d[Ca^{2+}]_{i}}{dt}=\beta'(I_{total}^{Ca}-I_{trop})$$

$\beta'=1/(1+\sum_{j} \frac{B_{\max,j}K_{d,j}}{([Ca^{2+}]_{i}+K_{d,j})^{2}})$ $j$: all intracellular Ca^2+^ buffers except for CaTRPN

$$I_{trop}=\frac{d[CaTRPN]}{dt}$$

$$\frac{d[CaTRPN]}{dt}=\frac{d[TCa]}{dt}+\frac{d[TCa*]}{dt}$$

$$\frac{d[T*]}{dt}=f1([Ca^{2+}]_{i},[T],[T*],[TCa],[TCa*])$$

$$\frac{d[TCa]}{dt}=f2([Ca^{2+}]_{i},[T],[T*],[TCa],[TCa*])$$

$$\frac{d[TCa*]}{dt}=f3([Ca^{2+}]_{i},[T],[T*],[TCa],[TCa*])$$

$$[T]=[Troponin]_{total}-[T*]-[TCa]-[TCa*]$$

, and are functions represent the transform among the four states ($[T],[T*],[TCa],[TCa*]$) in figure 2. See Equation (13) to (15) in Negroni_etal_1996 paper.

If the original model has dynamic equation for all buffers then there will be no instantaneous buffer factor β so simply ignore β in the implementation steps for this kind of model.

1. Initial conditions
2. and other variable values are quiescent values of the original model
3. are determined by solving $\frac{d[T*]}{dt}=0,\frac{d[TCa]}{dt}=0,\frac{d[TCa*]}{dt}=0$ .
4. $[\mathrm{CaTRPN}]=[\mathrm{TCa}]+[\mathrm{TCa}*]$, calculated from the above step, approximately equal to the value of $[\mathrm{CaTRPN}]$ in quiescent state if the original model has$[\mathrm{CaTRPN}]$.
5. There are multiple and in functions , $f2$ and : . Most of them are the same as original NL96 paper except for *Y1* and *Z1* which are chosen to match and in the one state CaTRPN buffer in either the original model ( if it has dynamic CaTRPN) or in Step 2.

**Implementation of instantaneous NL96 into models with instantaneous CaTRPN buffers**

1. Original NL96

$$\frac{dS_{j}}{dt}=f_{j}([Ca^{2+}]_{i},[T],[T*],[TCa],[TCa*])$$

$S_{j}=[T],[T*],[TCa],[TCa*]$ for $j=1,\cdots,4$

The ODE for $[T]$ can be replaced with the conservation law:

$$[T]+[T*]+[TCa]+[TCa*]=[\mathrm{Troponin}]_{total}$$

1. Instantaneous NL96

Set $\frac{dS_{j}}{dt}=f_{j}([Ca^{2+}]_{i},[T],[T*],[TCa],[TCa*])=0$ and solve for $[T],[T*],[TCa],[TCa*]$

The matrix form is $Ax=b$ :

$\exp o=\exp(-R(L-L_{a})^{2})$, see NL96 paper for detailed parameters and equations. At each time step solve $x=A^{-1}b$ to get $[T],[T*],[TCa],[TCa*]$

1. Original EP model with instantaneous buffers

$$\frac{d[Ca^{2+}]_{i}}{dt}=\beta I_{total}^{Ca}$$

$\beta=1/(1+\sum_{j} \frac{B_{\max,j}K_{d,j}}{([Ca^{2+}]_{i}+K_{d,j})^{2}})$ $j$: all intracellular Ca^2+^ buffers

1. Modified EP model with instantaneous NL96 implemented

$$\frac{d[Ca^{2+}]_{i}}{dt}=\beta'I_{total}^{Ca}$$

$$\beta'=1/(1+\sum_{j} \frac{B_{\max,j}K_{d,j}}{([Ca^{2+}]_{i}+K_{d,j})^{2}}+\frac{B_{TCa}K_{d,TCa}}{([Ca^{2+}]_{i}+K_{d,TCa})}+\frac{B_{TCa*}K_{d,TCa*}}{([Ca^{2+}]_{i}+K_{d,TCa*})})$$

$j$: all intracellular Ca^2+^ buffers except for CaTRPN

$B_{TCa}=[T]+[TCa]$, $B_{TCa*}=[T*]+[TCa*]$, $K_{d,TCa}=\frac{Z1}{Y1}$ $K_{d,TCa*}=\frac{Y3}{Z3}$

These parameters come from the two transitions involving $[Ca^{2+}]_{i}$
