# Supplementary material for: Implementation of Contraction to Electrophysiological Ventricular Myocyte Models, and Their Quantitative Characterization via Post-Extrasystolic Potentiation
Source: PLoS One. 2015 Aug 28;10(8):e0135699. doi: 10.1371/journal.pone.0135699 (PMC4552858; doi:10.1371/journal.pone.0135699)
Supplement: S2 File — (DOCX) [file pone.0135699.s002.docx]

S2: $[Ca^{2+}]_{i}$ transient for priming beat (steady state beat) for 14 models.
