# Supplementary material for: Implementation of Contraction to Electrophysiological Ventricular Myocyte Models, and Their Quantitative Characterization via Post-Extrasystolic Potentiation
Source: PLoS One. 2015 Aug 28;10(8):e0135699. doi: 10.1371/journal.pone.0135699 (PMC4552858; doi:10.1371/journal.pone.0135699)
Supplement: S4 File — (DOCX) [file pone.0135699.s004.docx]

S4: $MRC_{pes}$ for 14 models with NL96 contraction
