# Supplementary material for: Implementation of Contraction to Electrophysiological Ventricular Myocyte Models, and Their Quantitative Characterization via Post-Extrasystolic Potentiation
Source: PLoS One. 2015 Aug 28;10(8):e0135699. doi: 10.1371/journal.pone.0135699 (PMC4552858; doi:10.1371/journal.pone.0135699)

**S8: Comparisons between NL96 and two other contraction models**

**Comparison between NL96 and RWH99**

Since Iribe_etal_2006 has RWH99 in the original version, we compare the contraction dynamics between Iribe_etal_2006 with NL96 and with RWH99. Results of Iribe_etal_2006 with NL96 have been presented in previous figures while the results for Iribe_etal_2006 with RWH99 are in Fig S8.1 below. The two models show very similar characteristics except for two parameters: $t_{o,pes}$ and $T{}_{mrc,pes}$.

The PESI-axis interception $t_{o,pes}$ increases monotonically with respect to ESI for Iribe_etal_2006 with RWH99 (Fig S8.1 (e)) while it is not monotonic for NL96 (Fig 9 (b)), therefore its$MRC_{pes}$(Fig 6 (b)) curves cross each other at low PESI values. In addition, the $t_{o,pes}$ value range for NL96 is much lower than RWH99, whose value is closer to Yue’s experiment.

However, Iribe_etal_2006 with NL96 is more consistent with the experimental values of $T{}_{mrc,pes}$ compared to the corresponding RWH99 model (see Figure 10 (b) and Fig S8.1 (f)). The deviation in $T{}_{mrc,pes}$ is smaller for NL96 and more data points fall into Yue’s mean±SD range than RWH99.

Therefore, under the isometric conditions, we observed no significant differences between NL96 and RWH99 under the scope of this study in the sense that each model matches some parameters with experiments better than the other in this study.

**Comparison between NL96 and instantaneous NL96**

Including NL96 contraction into EP models with instantaneous or no $Ca^{2+}$ troponin buffers (Type Four and Five) changes the original $Ca^{2+}$ shape. To provide evidence that the deformed $Ca^{2+}$ transients are not the direct cause for the poor contraction behavior of this type of model, we introduce the ‘instantaneous NL96 contraction model’ by setting the dynamic equations in the NL96 model to be instantaneous, i.e. to set the right side of ODEs (Eq. (3)(4)(5)(6)) to be zero. In this way, all four states associated with the $Ca^{2+}$ troponin are instantaneous variables expressed by algebraic functions. Therefore we can easily incorporate them into EP models with instantaneous or no$Ca^{2+}$ troponin buffers (Type Four and Five). Detailed steps are shown in File S1. In this model’s construction, the time delay between $Ca^{2+}$ and the four states of $Ca^{2+}$ troponin buffer disappears. Figure S8.2 shows results after implementing the instantaneous NL96 into O’hara_etal_2011. Panel (a) is the priming $[Ca^{2+}]_{i}$ for O’hara_etal_2011 original (solid line), and with instantaneous NL96 (dash line). The systolic $[Ca^{2+}]_{i}$ is slightly elevated but the $[Ca^{2+}]_{i}$shape remains unchanged. Panel (b) shows the postextrasystolic potentiation in $Ca^{2+}$, of which the results are very similar to the original model. Panels (c) to panel (f) display the four contraction characteristic curves. They are similar to those of the O’hara_etal_2011 model with NL96 implemented (panel (e) of Figure 6, 8, 9 and 10) except that $MRC_{pes}$ and PESPC are shifted upward and $t_{o,pes}$ is shifted rightward due to the elevation of systolic $[Ca^{2+}]_{i}$. In a word, after the implementation of the instantaneous NL96, the $[Ca^{2+}]_{i}$ transient is hardly altered from the original model yet the contraction results are still not consistent with Yue’s experiment in multiple parameters.

**Fig S8.1.** **Dynamics of the Iribe_etal_2006 with RWH99 contraction:** (a) Priming$[Ca^{2+}]_{i}$; (b) postextrasystolic potentiation in$[Ca^{2+}]_{i}$; (c) postextrasystolic mechanical restitution curves ($MRC_{pes}$); (d) postextrasystolic potnetiation curve (PESPC); (e) minimum-value axis intercept curve ($t_{o,pes}$); (f) Time constant for $MRC_{pes}$ curve ($T_{mrc,pes}$).


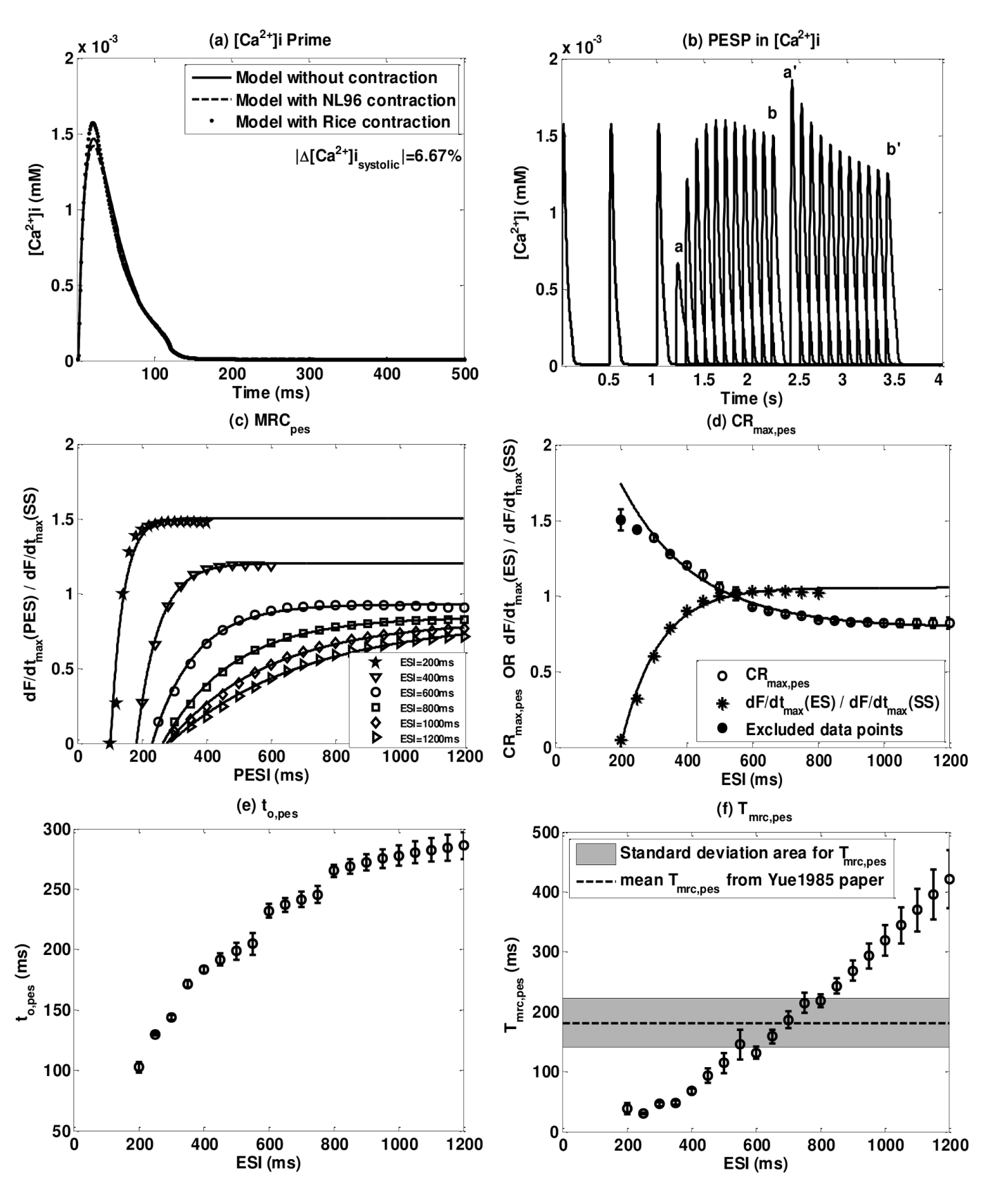


**Fig S8.2.** **Dynamics of the Ohara_etal_2011 with instantaneous NL96**: (a) Priming $[Ca^{2+}]_{i}$; (b) postextrasystolic potentiation in$[Ca^{2+}]_{i}$; (c) postextrasystolic mechanical restitution curves ($MRC_{pes}$ ); (d) postextrasystolic potnetiation curve (PESPC); (e) minimum-value axis intercept curve ($t_{o,pes}$); (f) Time constant for $MRC_{pes}$ curve ($T_{mrc,pes}$)
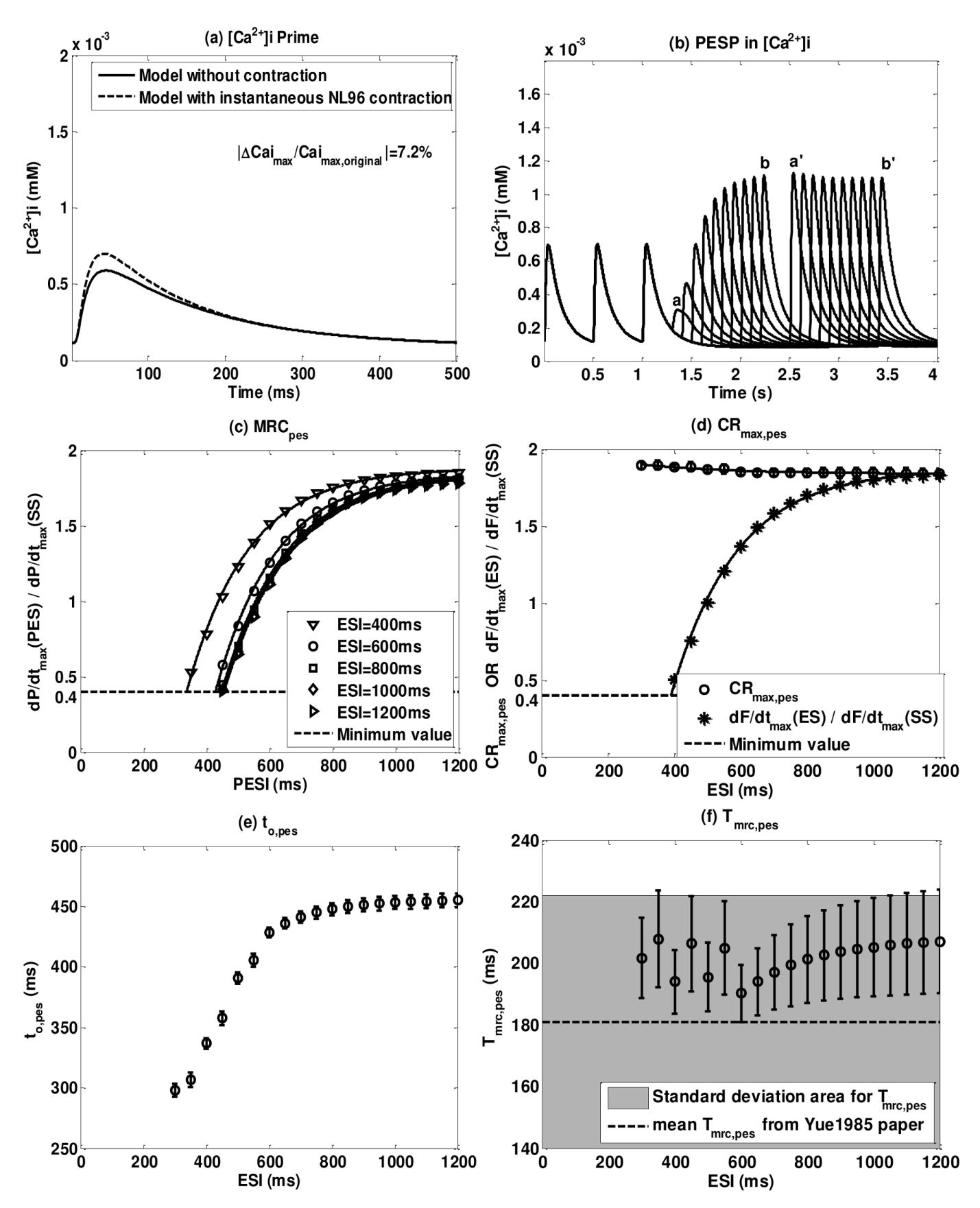

Supplement: S8 File — (DOCX) [file pone.0135699.s008.docx]
