# Supplementary material for: Implementation of Contraction to Electrophysiological Ventricular Myocyte Models, and Their Quantitative Characterization via Post-Extrasystolic Potentiation
Source: PLoS One. 2015 Aug 28;10(8):e0135699. doi: 10.1371/journal.pone.0135699 (PMC4552858; doi:10.1371/journal.pone.0135699)
Supplement: S9 File — (DOCX) [file pone.0135699.s009.docx]

**S9: Varying physiological parameters**

**Pacing Frequency**

In our studies we used 500ms as the priming pacing cycle length for all the models for two reasons. First, it’s close to the pacing period in Yue’s experiment[20], with which we compared our simulation results. Second, we wanted to compare all the models uniformly with one prime period (PP) and 500ms is close to the physiological heart rate of dogs and it’s between that of guinea pigs and humans. Nevertheless, to check and verify the validation of our results at 500 ms, we performed additional simulations on the Grandi_etal_2010 (human) model using 850ms as the priming period and the Iribe_etal_2006 (guinea pig) with PP=250ms. Figure S9.1 compares the $MRC_{pes}$, PESPC, $t_{o,pes}$, $T_{mrc,pes}$and the PESP in $[Ca^{2+}]_{i}$of the Grandi_etal_2010 model when using a pp=500ms (left panels) and 850ms (right panels). Similarly in Fig S9.2 we compare the $MRC_{pes}$ of the Iribe_etal_2006 model with PP=500ms (left panel) and 250ms (right panel). The Grandi_etal_2010 model shows qualitatively similar behaviors for 850ms comparing with 500ms and the difference in the amplitude of $MRC_{pes}$ and PESPC is directly related to the change in the normalization. The Iribe_etal_2006 model did not exhibit variations in the plateau values when the priming period is 250ms. On the other hand, this is mainly because Iribe_etal_2006 was not designed for fast pacing in the first. In the original Iribe paper [28] the priming period used is as long as 1200ms. It has been shown that actually most EP models breakdown and fail to reproduce physiological results at fast pacing. For example most fail to account for alternans and the few that do, tend to have much larger amplitudes and only for small ranges of CLs[1–3].

Therefore we believe that our results using uniform priming period of 500 ms for all models is a good compromise and does not change quantitatively the PESP behaviors when comparing with physiological pacing cycle length for different species.

**Temperature**

It has been shown that changes in temperature can produce large effects in the dynamics of cardiac tissue[4–7]. Because we are combining a room temperature contraction model (NL96) with body temperature (37°C) EP models, we wanted to investigate how temperature could influence our results. For this we reversed the approach of Matusoka_etal_2003 , where they modified the NL96 to physiological temperatures by multiplying the rate constants ($Y2,Z2,Y4,Yd$) by three[27]. We proceeded to change the rate constants in Matsuoka_etal_2003 back to the original values in NL96 and compare their dynamics. Figure S9.3 shows in panels (a) to (e) $MRC_{pes}$, PESPC, $t_{o,pes}$, $T_{mrc,pes}$ and the PESP in $[Ca^{2+}]_{i}$for the Matsuoka_etal_2003 model with physiological temperature (left panels) and with room temperature (right panels). We observed that changing the rate constants back to room temperature values did not change any of the four contraction characteristic curves or the $[Ca^{2+}]_{i}$. This shows that combining room temperature NL96 contraction to body temperature EP models should not affect the results obtained in this study. However it is important to notice that most cell models do not produce large changes when temperatures are modified[8] and thus models need to be tuned to experimental data[6,7]

**Cooperativity**

The NL96 has limited cooperativity as the Hill coefficient used in simulations is 1.3 which is lower than reported in experiments (between 2 and 6)[9][10]. Negroni and Lascano published a newer version of their contraction model NL08[10], with two major changes. First they used a troponin system (TS), which consists of three adjacent troponin-tropomyosin regulatory units as the basic unit; second, the cross-bridges have two possible conformations: strong and weak, which results in six total states for the troponin system instead of four. The newer model could reproduce better cooperative effect required for force-$Ca^{2+}$ relationships and force response to length steps. The Hill coefficient is calculated to be from 4.15 to 4.00, corresponding to L=0.9 to 1.1µm[10]. To investigate the influence of the Hill coefficient, we implemented NL08 into Matsuoka_etal_2003 and compared it with the original Matsuoka model (with NL06). Results are shown in Fig S9.3. We found no notable difference in the four contractile characteristic curves (subfigure (a) to (d)) between NL96 (left panels) and NL08 (right panels). The postextrasystolic potentiation in $Ca^{2+}$ is also retained (subfigure (e)) except for the reduced maximum value of $[Ca^{2+}]_{i}$. The Hill coefficient mainly determines the absolute value of force as a function of Calcium concentration. Since the $MRC_{pes}$ and PESPC are normalized (to the prime beat) force changing rate, the contraction characteristic curves are maintained after the alteration of Hill coefficient.

Figure S9.1. Grandi_etal_2010 model with priming period of 500ms (left panels) and 850ms (right panels): (a)$MRC_{pes}$, (b) PESPC, (c) $t_{o,pes}$, (d) $T_{mrc,pes}$ (e) PESP in $[Ca^{2+}]_{i}$.


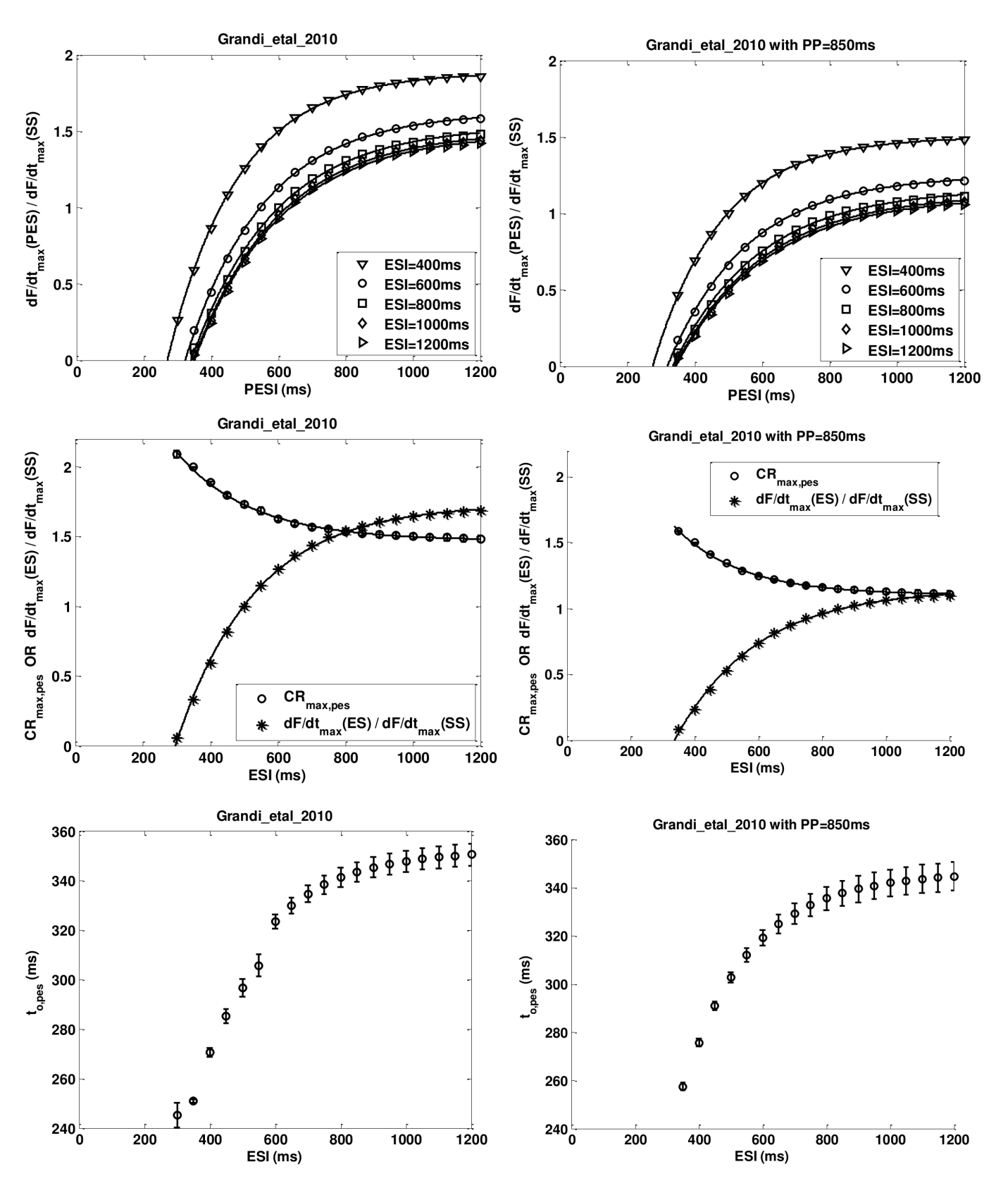


(c)

(b))

(a)


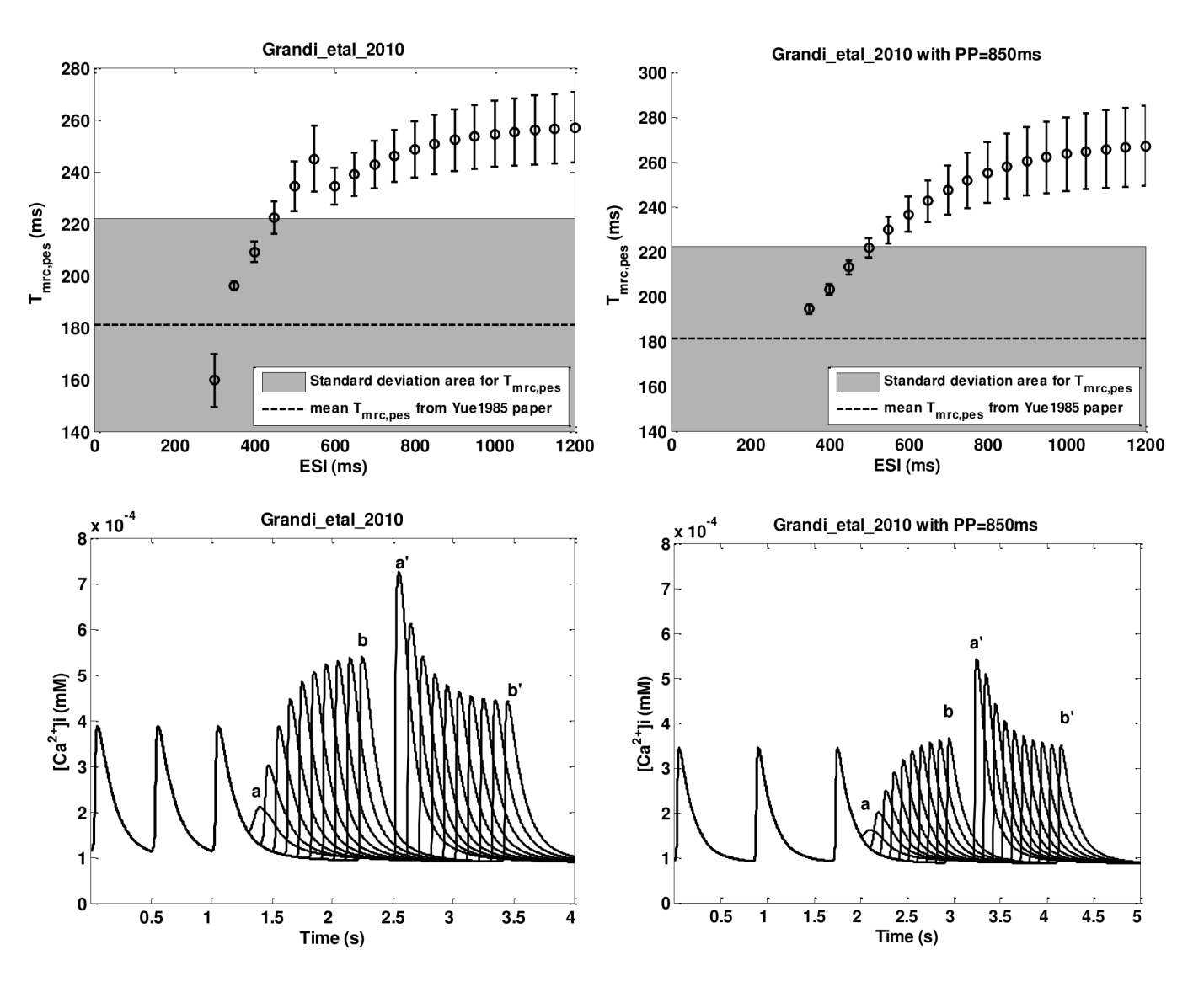


(e)

(d)

Figure S9.2. Matsuoka_etal_2003 model of body temperature parameters (left) and room temperature parameters (right): (a)$MRC_{pes}$, (b) PESPC, (c) $t_{o,pes}$, (d) $T_{mrc,pes}$ (e) PESP in $[Ca^{2+}]_{i}$.
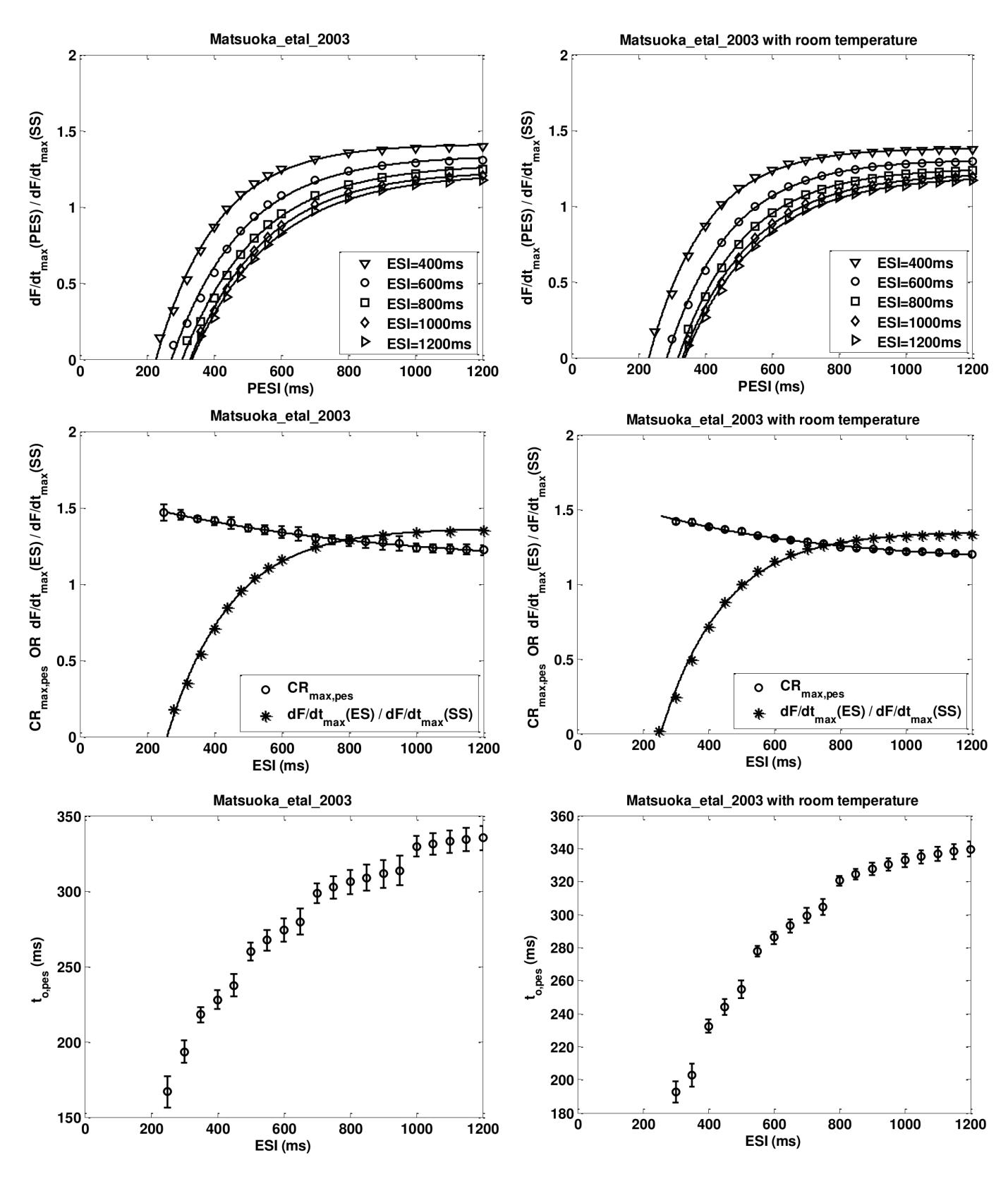


(c)

(b)

(a)


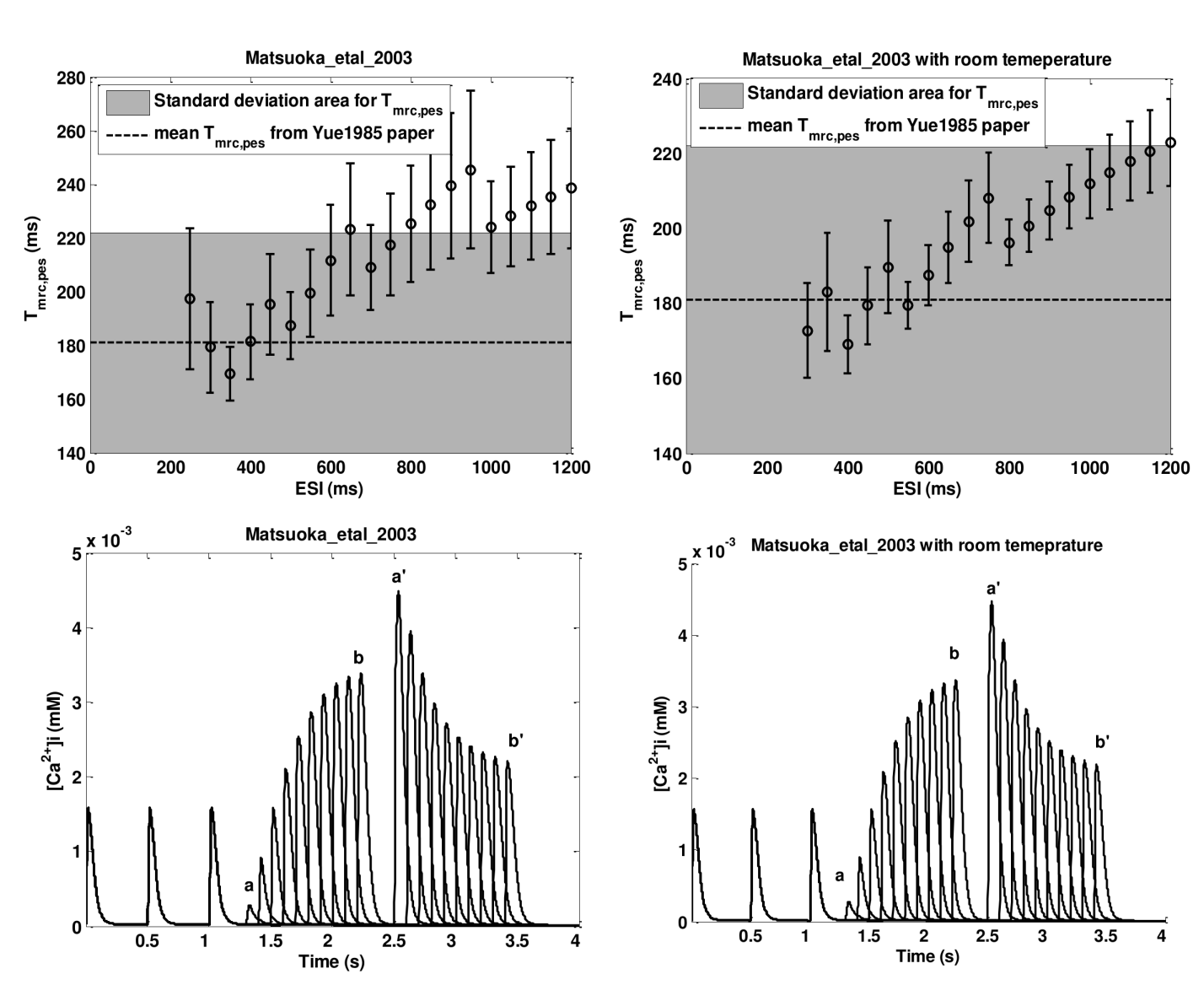


(e)

(d)

Figure S9.3. Matsuoka_etal_2003 model with NL96 contraction (left) and NL08 contraction (right): (a)$MRC_{pes}$, (b) PESPC, (c) $t_{o,pes}$, (d) $T_{mrc,pes}$ (e) PESP in $[Ca^{2+}]_{i}$.


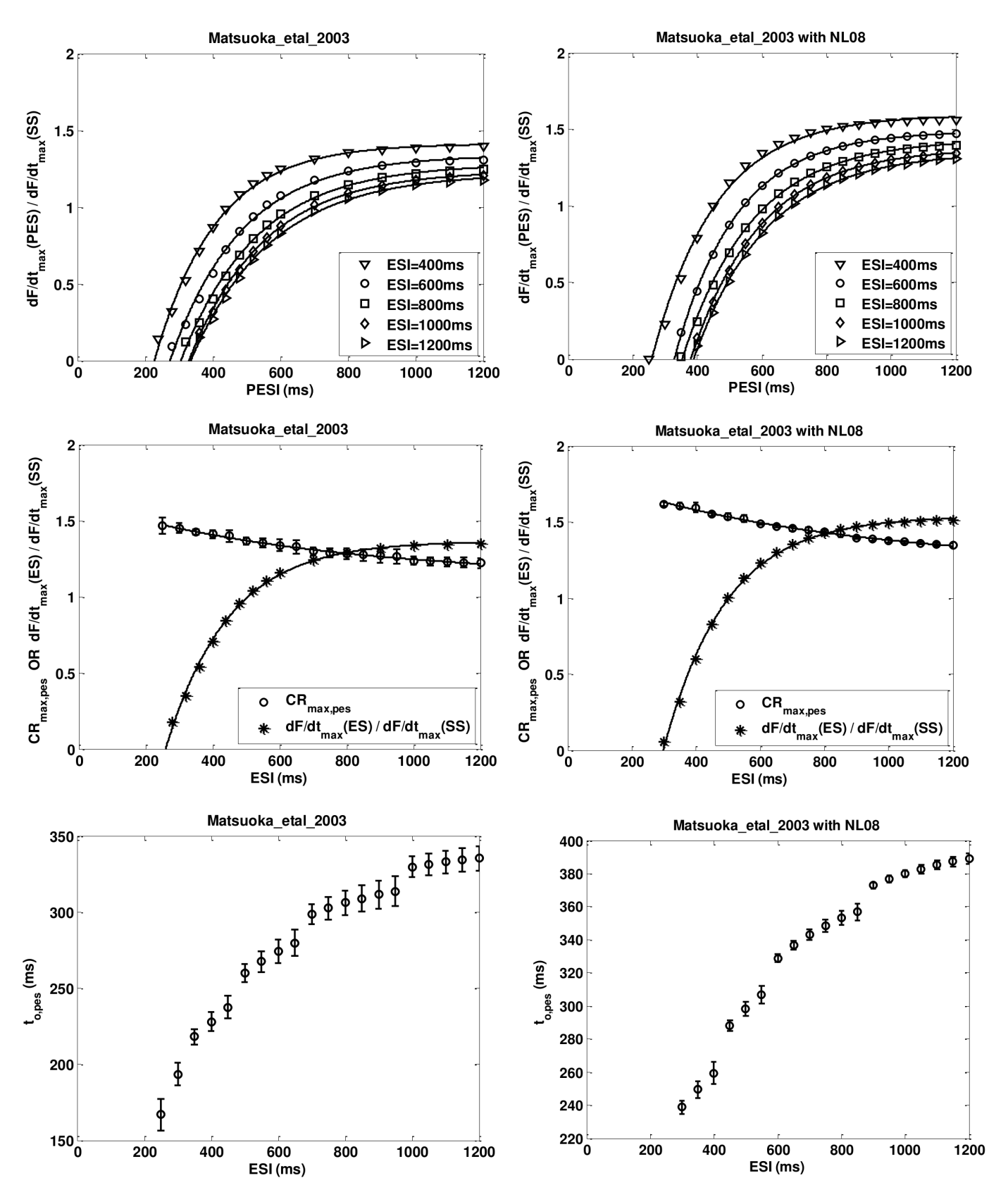


(c)

(b)

(a)


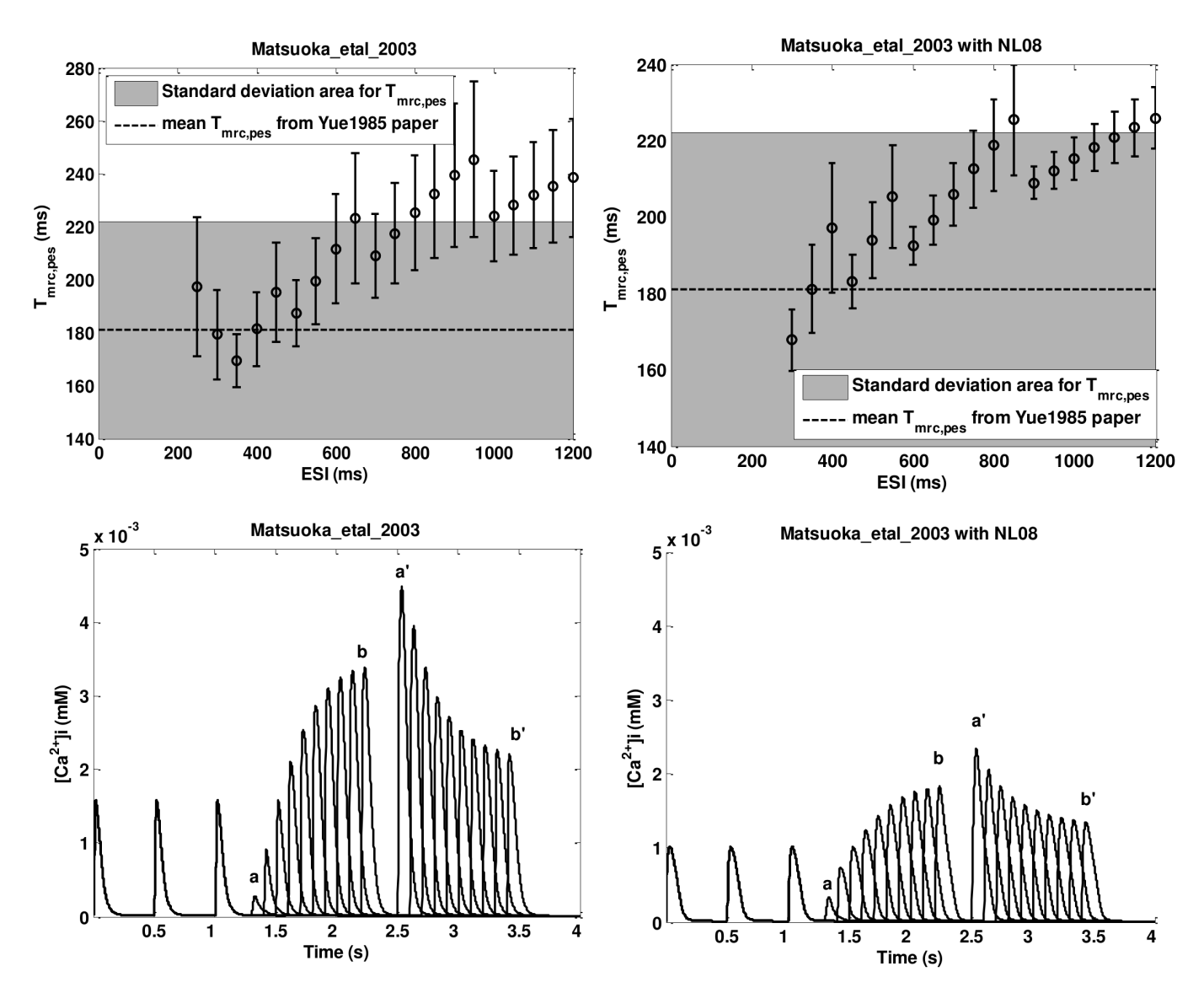


(e)

(d)

**Reference**

1. Cherry EM, Fenton FH. A tale of two dogs: analyzing two models of canine ventricular electrophysiology. Am J Physiol Heart Circ Physiol. 2007;292: H43–55. doi:00955.2006

2. Clayton RH, Bernus O, Cherry EM, Dierckx H, Fenton FH, Mirabella L, et al. Models of cardiac tissue electrophysiology: progress, challenges and open questions. Prog Biophys Mol Biol. 2011;104: 22–48.

3. Cherry EM, Fenton FH, Gilmour RF Jr. Mechanisms of ventricular arrhythmias: a dynamical systems-based perspective. Am J Physiol Heart Circ Physiol. 2012;302: H2451–2463. doi:10.1152/ajpheart.00770.2011

4. Templeton GH, Wildenthal K, Willerson JT, Reardon WC. Influence of Temperature on the Mechanical Properties of Cardiac Muscle. Circ Res. 1974;34: 624–634. doi:10.1161/01.RES.34.5.624

5. Langer GA, Brady AJ. The Effects of Temperature upon Contraction and Ionic Exchange in Rabbit Ventricular Myocardium. J Gen Physiol. 1968;52: 682–713.

6. Fenton FH, Gizzi A, Cherubini C, Pomella N, Filippi S. Role of temperature on nonlinear cardiac dynamics. Phys Rev E. 2013;87: 042717. doi:10.1103/PhysRevE.87.042717

7. Filippi S, Gizzi A, Cherubini C, Luther S, Fenton FH. Mechanistic insights into hypothermic ventricular fibrillation: the role of temperature and tissue size. Eur Eur Pacing Arrhythm Card Electrophysiol J Work Groups Card Pacing Arrhythm Card Cell Electrophysiol Eur Soc Cardiol. 2014;16: 424–434. doi:10.1093/europace/euu031

8. Fink M, Niederer SA, Cherry EM, Fenton FH, Koivumäki JT, Seemann G, et al. Cardiac cell modelling: observations from the heart of the cardiac physiome project. Prog Biophys Mol Biol. 2011;104: 2–21.

9. Kentish JC, ter Keurs HE, Ricciardi L, Bucx JJ, Noble MI. Comparison between the sarcomere length-force relations of intact and skinned trabeculae from rat right ventricle. Influence of calcium concentrations on these relations. Circ Res. 1986;58: 755–768.

10. Yue DT, Marban E, Wier WG. Relationship between force and intracellular [Ca2+] in tetanized mammalian heart muscle. J Gen Physiol. 1986;87: 223–242. doi:10.1085/jgp.87.2.223

10. Negroni JA, Lascano EC. Simulation of steady state and transient cardiac muscle response experiments with a Huxley-based contraction model. J Mol Cell Cardiol. 2008;45: 300–312. doi:10.1016/j.yjmcc.2008.04.012
